# Natural Negative Feedback Loops Confer *Indica*‐*Japonica* Differentiation for Grain Size Homeostasis in Rice

**DOI:** 10.1002/advs.202516180

**Published:** 2026-02-04

**Authors:** Xingxing Li, Meng‐en Wu, Ziqi Qiao, Junkui Huang, Juncheng Zhang, Yang Ding, Junqing Zhu, Jingyue Xu, Yuxin Huang, Wei Li, Xiaomin Su, Yue Ding, Jianwei Zhang, Yibo Li

**Affiliations:** ^1^ National Key Laboratory of Crop Genetic Improvement and National Centre of Plant Gene Research (Wuhan) Huazhong Agricultural University Wuhan China; ^2^ Hubei Hongshan Laboratory Wuhan China

**Keywords:** grain size homeostasis, indica‐japonica differentiation, natural negative feedback loop, rice

## Abstract

Organ size homeostasis plays vital roles in maintaining the normal growth and development in both animals and plants. Grain size is an important agronomic trait for stable yield, quality, domestication, and breeding in crops, but the molecular mechanism underlying final size homeostasis remains unclear. Here, we identified three genes, *OsGRX8*, *OsbZIP47* and *OsbZIP08*, underlying grain‐length variation by genome‐wide association study (GWAS) in rice. We confirmed that OsGRX8, OsbZIP47 and OsbZIP08 interact with each other and transcription factors OsbZIP47 negatively and OsbZIP08 positively regulate the expression of the downstream glutaredoxin‐encoding gene *OsGRX8*. The binding ability of OsbZIP08 on the promoter of *OsGRX8* in *indica* is higher than that in *japonica*, leading the differential expression of *OsGRX8* between two subspecies. We further revealed a natural negative feedback regulatory mechanism for grain size homeostasis: OsGRX8 controls the reduction modification of OsbZIP47 thereby increasing OsbZIP47‐OsbZIP08 interaction in a redox‐dependent way or directly interacts with OsbZIP08 in a redox‐independent way to inhibit the transcriptional activity of OsbZIP08 on *OsGRX8*. Finally, we revealed that two self‐regulatory haplotypes (SRHs), caused by co‐selected variations of the three genetically unlinked genes which formed the negative feedback loops, showed distinctive *indica*‐*japonica* differentiation and large genetic contribution to key yield traits. Our findings provided the evolutional OsGRX8‐(OsbZIP47)‐OsbZIP08‐*OsGRX8* regulatory loops for synergistically controlling grain size homeostasis by fine‐tuning *OsGRX8* self‐expression, offering a novel case for uncovering QTL interactions underlying genetic diversity of important traits in crops.

## Graphical abstract

This study reveals the genetic and molecular mechanisms controlling grain size homeostasis through fine‐tuning *OsGRX8* self‐expression by two natural negative feedback loops functioning in redox‐dependent or ‐independent manners and identifies two self‐regulatory haplotypes (SRHs) for the subspecies differentiation in rice. This work holds significant implications for a self‐regulation mechanism of organ size homeostasis by uncovering QTL interactions in crops.

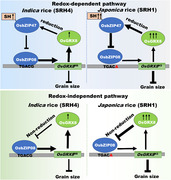

## 1 Introduction

Organ size homeostasis is an intrinsic and basic feature to ensure the proper size and shape of tissues and organs, which is very important for maintaining their normal functions and self‐organizing processes [1, 2, 3, 4]. Grain shape/size in rice is highly heritable thus it is one of the ideal models for studying organ shape development and organ size homeostasis in plants [5, 6]. However, the regulatory mechanism of organ/grain size homeostasis in plants is unknown. Grain size controls stable grain yield, appearance, and milling quality in rice, but its genetic basis is very complex as it is controlled by multiple quantitative trait loci (QTL) genes (QTGs) [7, 8]. A number of QTL or genes controlling rice grain size have been identified [9], and most of them were involved in several signaling pathways, including IAA, CK, and BR biosynthesis and signaling pathway, G protein signaling, MAPK signaling, the ubiquitin‐proteasome degradation pathway, epigenetic pathways, peptide signaling, and transcriptional regulation [5, 10, 11]. Although a large number of QTGs for an important agronomic trait have been identified and cloned, the interaction mechanism among these QTGs is poorly understood, which makes it too difficult to understand the molecular mechanisms underlying natural genetic diversity and improve the breeding efficiency in crops [5, 12]. Furthermore, grain size is an important domestication trait in crops, especially for two rice subspecies, *indica* and *japonica*. Thus, it is needed to explain the *indica*‐*japonica* differentiation and artificial selection signatures for grain size breeding.

Redox homeostasis is an essential and dynamic process and accounts for a healthy physiological steady state by regulating oxidative eustress, which is precisely balanced by the generation and elimination of redox reactions [13, 14, 15, 16, 17]. Glutaredoxins (GRX) are small thiol proteins of the thioredoxin family and key players in the maintenance of cellular redox homeostasis, due to their unique characteristic of catalyzing a reversible reduction for glutathione‐dependent redox modifications through glutathionylation and deglutathionylation [15, 18]. GRXs play a prominent role in regulating vital physiological, developmental, abiotic, and biotic stress processes [19, 20], however the redox homeostasis mechanism maintained by GRXs in plants remains unclear. The OsGRX8/WG1 in rice and its homologous proteins ROXY1/2 in Arabidopsis and MSCA1 in maize were classified into the CC‐type GRXs with a plant‐specific CCXC motif [21, 22, 23, 24]. OsGRX8/WG1 interacts with GS3 and reduces the oligomerization of GS3, which inhibits the interaction between RGB1 and DEP1/GGC2 for short grains [25]. In addition, the TGA/bZIP transcription factors, such as OsbZIP47 and OsbZIP08 in rice, FEA4 in maize, and PAN in Arabidopsis, participate in biotic stress response, development, detoxification, and flowering processes [26]. GRXs interact with TGA/bZIP transcription factors, which are conserved in Arabidopsis, maize, and rice [23, 27, 28, 29]. For example, OsGRX8/WG1 interacts with OsbZIP47 and recruits the transcriptional co‐repressor ASP1 to inhibit the transcriptional activity in regulating grain width in rice [28]. Despite the important regulatory roles of the protein complex OsGRX8‐OsbZIP47 in various developmental processes, natural variations of these genes have not been discovered and how seed size homeostasis is determined by redox homeostasis remains elusive in rice [26, 30].

The most fundamental function of negative feedback regulation is to coordinate the homeostasis of biological process or optimize the activity of a protein, enabling the maintenance of a stable and beneficial status [31, 32, 33, 34]. For example, the stem cells in SAM are maintained by the CLAVATA‐WUSCHEL negative feedback loop [35, 36]. A negative feedback loop of TOR signaling in sugar sufficiency balances the trade‐offs between nutrient‐dependent growth and stress in plants [37, 38]. However, in rice, the molecular mechanism underlying the self‐regulation of a gene remains largely unclear.

Here we identified the natural variations of three genes *OsGRX8*, *OsbZIP47*, and *OsbZIP08* that control grain‐length variation through GWAS in rice, and verified the interactions among the three QTGs genetically and biochemically. We further revealed natural negative feedback loops: OsGRX8 negatively regulates its self‐expression through promoting the interaction of OsbZIP47 and OsbZIP08 or directly binding with OsbZIP08, subsequently inhibiting the transcriptional activity of OsbZIP08 on *OsGRX8*. We therefore identified the negative feedback pathway of OsGRX8‐(OsbZIP47)‐OsbZIP08‐*OsGRX8* confers *indica*‐*japonica* differentiation for grain size homeostasis in rice. Furthermore, the self‐regulatory haplotypes (SRHs) caused by co‐selected natural variations among the three genes were identified and showed distinctive geographical distribution, *indica*‐*japonica* differentiation, and artificial selection signatures.

## 2 Results

### 2.1 Natural Variations in *OsGRX8* Control Grain Length Diversity between Rice *Indica‐* *Japonica* Subspecies

**FIGURE 1:**
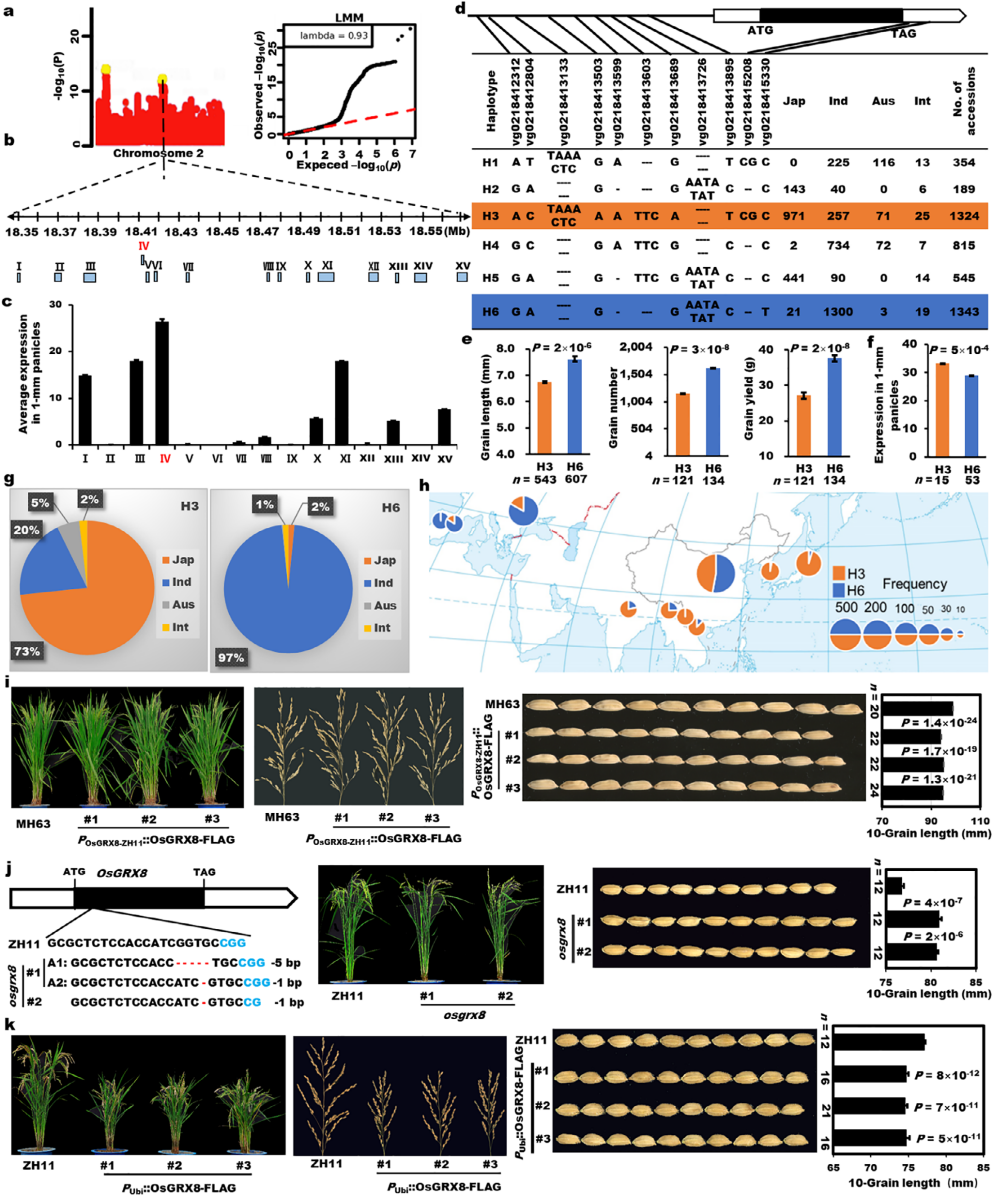
Natural variations of *OsGRX8* regulate grain size variation between rice subspecies. (a) Manhattan and quantile‐quantile plots for GWAS of grain length on chromosome 2. (b) The underlying candidate genes in the peak region. The red dotted line indicates the most significant QTL containing the *OsGRX8* gene. (c) Average expression levels of candidate genes in the 1‐mm panicle of 533 accessions. (d) The haplotypes and their subspecies distribution of *OsGRX8* based on their representative variations in 4726 accessions. (e) Grain length, grain number, and grain yield of the two major haplotypes. (f) The expression levels of the two major haplotypes in 1‐mm panicles of 271 accessions. (g) The *indica*‐*japonica* differentiation of the major haplotypes of H3 and H6. (h) Geographical distribution of H3 and H6 using 4726 accessions. The figure is based on the standard map with the ID GS(2016)2962 that was taken from the website of the National Platform for Common Geospatial Information Services. (i) Plant architecture, panicle length, and grain length of *P*_*OsGRX8*‐ZH11_::*OsGRX8*‐FLAG complementation lines in T_2_ progenies. (j) The sgRNA target site for generating the *osgrx8* mutants by CRISPR/*Cas9* in ZH11 and the plant architecture and grain‐length phenotype of them. PAM sequences are marked in blue. (k) Plant architecture, panicle morphologies, and grain length of *P*_*Ubi*_::*OsGRX8*‐FLAG lines. *n* is the number of accessions of each haplotype or individuals of each transgenic line. All data are shown as mean value ± SEM. All the *p* values were produced by two‐tailed *t*‐tests.

To identify novel QTGs for grain size regulation in rice, GWAS was performed using grain length as an indicator trait of a mini‐core collection of 533 accessions worldwide, whose population structure was analyzed using the principal component analysis (PCA) plot and kinship matrix heatmap (Figure S1 and Table S1). A significant QTL on chromosome 2 was detected for grain length by the linear mixed model in the GWAS using all the 533 accessions (Figure 1a; Figure S2a). Fifteen possible candidate genes within 200 kb at the peak single nucleotide polymorphism (SNP) site for grain length were identified (Figure 1b). Eliminating transposons, retrotransposon and some genes with known functions, the gene IV (*LOC_Os02g30850*, *OsGRX8*, encoding a CC‐type glutaredoxin) predominantly expressed in the 1‐mm and 4‐cm young panicles was identified (Figure 1c; Figure S2b and Table S2). *OsGRX8* was cloned by reverse genetics to regulate grain length, grain width and weight in rice [25, 28]，thus, it is probably the causal gene underlying the QTL.

To characterize the natural variation effect of *OsGRX8* on grain length, we performed in‐depth haplotype analysis using 4726 rice accessions worldwide [39, 40] and identified six haplotypes of *OsGRX8* (H1–H6) based on the representative natural variations (Figure 1d; Table S3). In addition, there are obvious differences between the two major haplotypes (H3 and H6) in the grain length of 2013 accessions, and H3 showed the higher expression than that of H6 in 1‐mm panicles of 271 accessions (Figure 1e,f), showing the potential effect of these variations on *OsGRX8* transcriptional regulation. H3 was mainly present in *japonica*/*Geng* accessions (73%), while H6 were mainly distributed in the *indica*/*Xian* accessions (97%) (Figure 1g). Furthermore, we analyzed the representative natural variations in the *OsGRX8* promoter and found that the most significant variation of vg0218413503 can significantly distinguish the expression level of *OsGRX8* in young panicles, grain length, grain yield, and grain width traits in 530 accessions (Figure S2c,d). So, the vg0218413503 variant site (A/G) maybe functions as an important variation for *OsGRX8* in rice. Furthermore, haplotype network analysis of *OsGRX8* using the representative variations in 4726 accessions suggested that *OsGRX8* contributes greatly to *indica*‐*japonica* differentiation (Figure S2e). Subsequently, the geographical distribution of H3 and H6 showed that H3 was mainly found in Southeast Asia and East Asia, while H6 was predominantly found in Europe (Figure 1h), inferring that *OsGRX8* shows obvious geographical distribution differentiation.

To confirm the grain length difference of the two major haplotypes, we constructed the complementation transgenic lines for *OsGRX8* by introducing the stronger allele from the H3 representative *japonica* variety ZH11 into the H6 representative *indica* variety MH63 with the weaker allele. The three positive complementation lines of *OsGRX8* showed shorter grains than the wild type, despite of the same phenotypes of both plant architecture and panicle length (Figure 1i). To confirm the function of *OsGRX8* in regulating grain length in vivo, we obtained various knockout mutants of *OsGRX8* in ZH11, the stronger function background of the H3 haplotype (Figure 1j). Although plant height, panicle length, and tiller number had no difference between ZH11 and *osgrx8* mutants, the grains of positive plants were apparently longer than wild type (Figure 1j; Figure S3a). In addition, after mutating its homologous genes *OsGRX13*/*24*/*26* in ZH11 (Figure S3b, c), we discovered that *osgrx8/13* and *osgrx8/13/24/26* mutants exhibited similar phenotypes with *osgrx8* mutants (Figure S3d,e). To further verify the genetic effect of *OsGRX8*, we also generated *OsGRX8* overexpression lines, and the plant height, panicle, and grain length had a significant decrease but the tiller number increased, compared with wild type (Figure 1k; Figure S3f), which was confirmed by the co‐segregation tests of grain length and panicle length (Figure S3g). These results indicated that natural variations of *OsGRX8* control grain length variation between two rice subspecies.

### 2.2 Natural Variations in *OsbZIP47* Confer *Indica*‐*japonica* Differentiation for Grain Length in Rice

**FIGURE 2:**
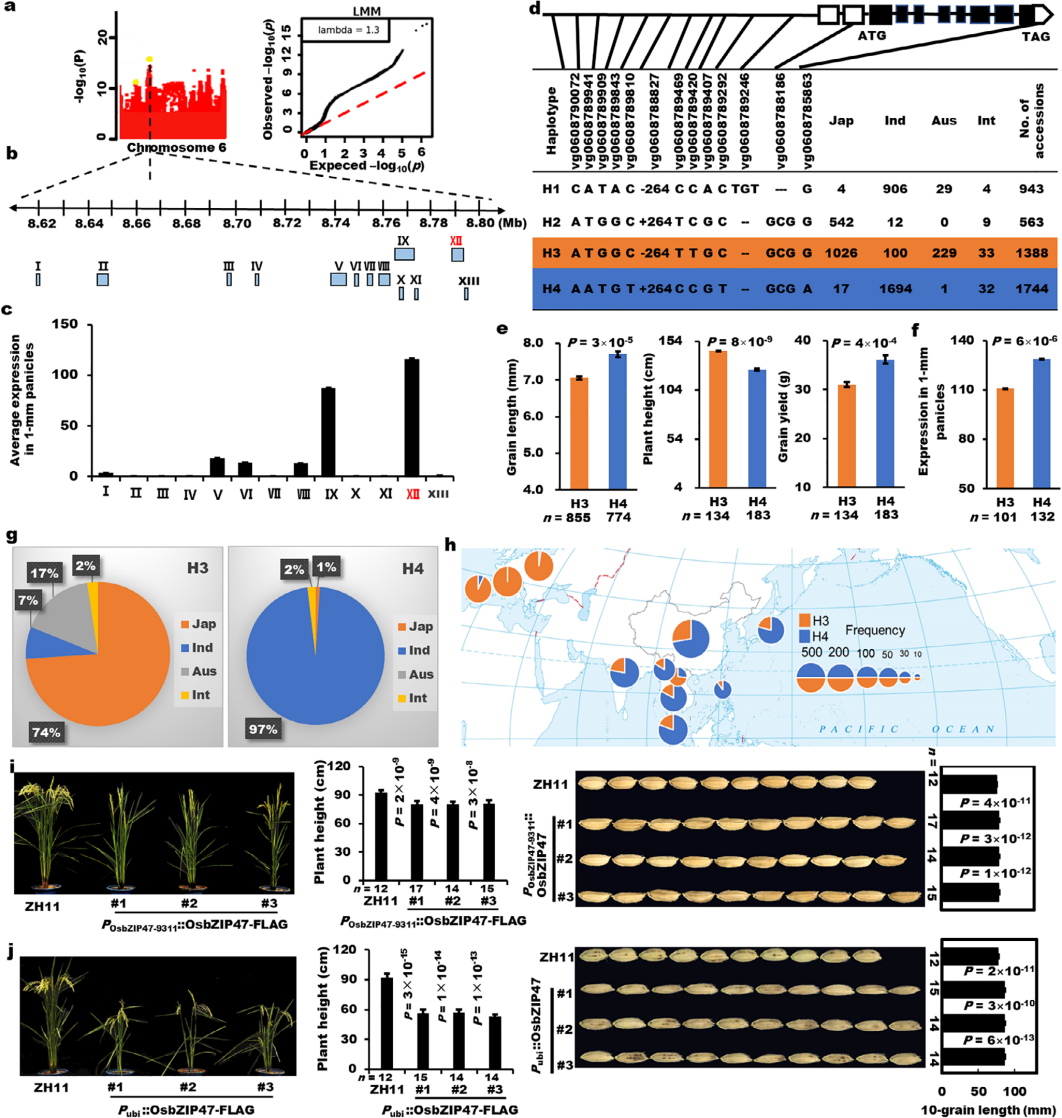
Natural variations in *OsbZIP47* confer grain length variation between *indica*‐*japonica* subspecies. (a) Manhattan and quantile‐quantile plots for GWAS of grain length. (b) Possible functional genes and their positions within 200 kb around the peak point. (c) Average expression levels of candidate genes in 1‐mm panicle of 533 accessions. (d) Major representative natural variations of *OsbZIP47* and its four haplotypes in 4726 accessions. (e) Grain length, plant height, and grain yield of two major haplotypes (H3 and H4). (f) Expression levels between the two major haplotypes in 1‐mm panicles of 271 accessions. (g) *Indica*‐*japonica* differentiation of the major haplotypes of H3 and H4. (h) Geographical distribution of H3 and H4 using 4726 accessions. The source of the figure is the same to Figure 1h. (i,j) Plant height and grain length of three *P_OsbZIP47_*_‐9311_::*OsbZIP47*‐FLAG complementation lines (i) and *P*_*Ubi*_::*OsbZIP47*‐FLAG materials (j) in T_1_. *n* is the number of accessions of each haplotype or the number of individuals of each transgenic line. All data are shown as mean value ± SEM. All the *p* values were produced by two‐tailed *t*‐tests.

Another significant peak point on chromosome 6 was identified by GWAS using 533 accessions with *GW5* as the covariate (Figure 2a; Figure S4a). Eliminating transposons, retrotransposon and some known grain size genes, 13 possible candidate genes were identified (Figure 2b). A gene *LOC_Os06g15480* (*OsbZIP47*), encoding a bZIP transcription factor, located in the peak point region and supported by a high expression pattern in 1‐mm and 4‐cm panicles, was identified as a solid candidate gene of the locus (Figure 2c; Figure S4b and Table S4).

To explore the natural variations of *OsbZIP47*, we performed in‐depth haplotype and geographical distribution analysis using 4726 accessions. Thirteen major representative variations out of all 39 natural variations in the promoter and coding region of *OsbZIP47* were identified and classified into four haplotypes (H1–H4) (Figure 2d; Table S5). The two major haplotypes (H3 and H4) can significantly distinguish grain length in 2013 accessions and plant height and grain yield in 533 accessions (Figure 2e). The grain size trend of the two major haplotypes positively co‐related with *OsbZIP47* expression level in 1‐mm panicles (Figure 2f). Furthermore, despite of the most significant variation (A/C) of vg0608790072, the vg0608788827 containing a 264‐bp InDel located in the chromatin accessible region, can significantly distinguish expression level of *OsbZIP47*, grain length, grain yield and grain width traits in 533 accessions (Figure S4c–e), suggesting that the 264‐bp InDel may function as the important variation for *OsbZIP47* in rice.

H3 and H4 had absolute advantages in the *japonica/Geng* (74%) and *indica/Xian* (97%) subspecies, respectively, as the similar result in haplotype network analysis of *OsbZIP47* using the representative variations in 4726 accessions (Figure 2g; Figure S4f). Subsequently, the geographical distribution of H3 and H4 showed that H3 was mainly found in Europe, while H4 was predominantly found in Southeast Asia and East Asia (Figure 2h). All the results suggested that *OsbZIP47* could contribute greatly to *indica*‐*japonica* differentiation and its main haplotypes show obvious geographical distribution difference.

To confirm the function difference in grain length between two major haplotypes, we constructed the complementation transgenic lines for *OsbZIP47* by introducing the stronger allele from the H4 representative *indica* variety 9311 into the H3 representative *japonica* variety ZH11 with the weaker allele. Three independent positive transgenic lines of T_1_ exhibited increased grain length and decreased plant height as well as panicle length compared to wild type (Figure 2i; Figure S5a). Secondly, three positive over‐expression lines of *OsbZIP47* exhibited longer grains and decreased plant height, panicle length, and tiller number (Figure 2j; Figure S5b). Furthermore, the co‐segregation tests between genotype and grain length phenotype in T_1_ progenies of the complementation and over‐expression lines confirmed the results (Figure S5c). Because of the weak allele feature in the background of ZH11, the CRISPR lines with different mutations generated in ZH11 showed no difference in grain length, plant height, and panicle length compared with wild type (Figure S5d, e). Taken together, these results confirmed that natural variations of *OsbZIP47* control the variation of grain size, plant height, panicle length, and tiller number between rice subspecies.

### 2.3 Contribution of Natural Variations in *OsbZIP08* to *indica*‐*japonica* Differentiation and Grain Size Diversity

**FIGURE 3:**
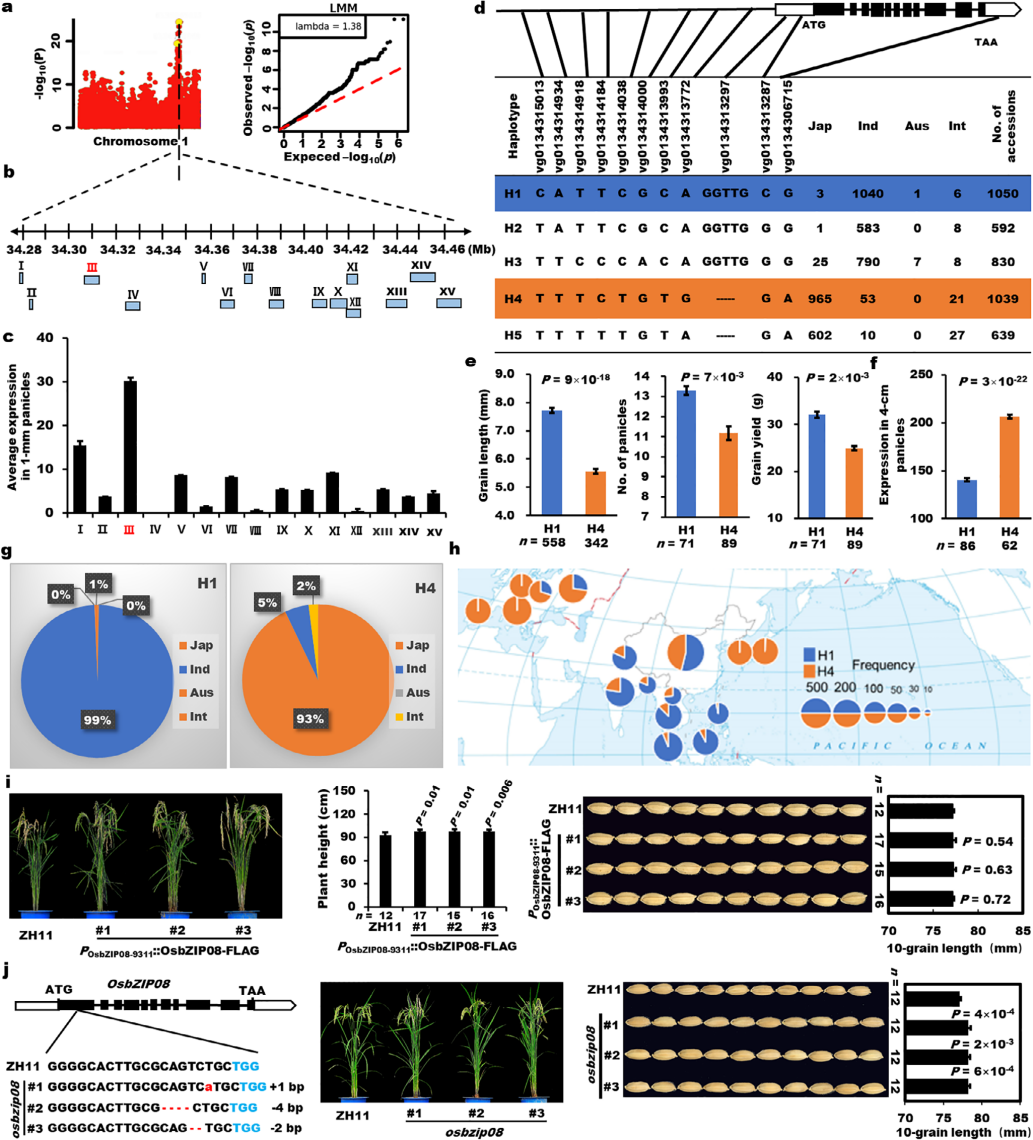
*OsbZIP08* is identified by GWAS for grain length variation underlying *indica*‐*japonica* differentiation in rice. (a) The GWAS locus on chromosome 1 detected for rice grain length. (b) Location of the predicted ORFs around the peak point. (c) Average expression levels of candidate genes in 1‐mm panicles of the mini‐core collection. (d) Major haplotypes of *OsbZIP08* based on the representative natural variations and their subspecies distribution in 4726 accessions. (e) Grain length in 2013 accessions, number of panicles, and grain yield in 533 accessions of the major haplotypes of H1 and H4. (f) Expression levels between the two major haplotypes in 4‐cm panicles in 265 accessions. (g) The *indica*‐*japonica* differentiation of H1 and H4. (h) The geographical distribution of H1 and H4 using 4726 accessions. The source of the figure is the same to Figure 1h. (i) Plant architecture and grain shape of the *P_OsbZIP08_*_‐9311_::*OsbZIP08*‐FLAG complementation lines. (j) Plant height and grain length of *osbzip08* mutants obtained by CRISPR. PAM sequences are marked in blue. *n* is the number of accessions of each haplotype or the number of individuals of each transgenic line. All data are shown as mean value ± SEM. All the *p* values were produced by two‐tailed *t*‐tests.

A significant locus for grain size was identified on chromosome 1 by GWAS for grain length using temperate *japonica* in 533 accessions (Figure 3a; Figure S6a). The 15 predicted genes within the 200‐kb interval around the peak SNP were carefully analyzed (Figure 3b). The expression levels of these genes in 1‐mm and 4‐cm young panicles showed that *LOC_Os01g59350* (*OsbZIP08*) exhibited the highest expression level in young panicles (Figure 3c; Figure S6b and Table S6). Therefore, *OsbZIP08*, encoding a bZIP transcription factor, was considered as the candidate gene of the locus.

To examine whether *OsbZIP08* is associated with grain size in the natural population of 4726 accessions, we identified five haplotypes classified by the 11 representative variations out of all 30 natural variations in *OsbZIP08* (Figure 3d; and Table S7). There were significant differences in grain length, panicle number, and grain yield between the two major haplotypes (H1 and H4) (Figure 3e), with the higher expression of *OsbZIP08* in H4 than that in H1 (Figure 3f). Furthermore, we identified the important variation in the promoter of *OsbZIP08* in a similar way as above. The vg0134314934 variation (A/T) located in the chromatin accessible region can significantly distinguish grain length, plant height, and 1000‐grain weight traits, which are co‐related with *OsbZIP08* expression level (Figure S6c, d). Thus, this A/T variation may function as a significant variation of *OsbZIP08* in rice.

The haplotype cluster analysis of *OsbZIP08* among the 4726 accessions suggested that *OsbZIP08* could contribute to *indica*‐*japonica* differentiation (Figure S6e). Furthermore, H1 and H4 of *OsbZIP08* mainly consists of *indica/Xian* (99%) and *japonica/Geng* (93%) accessions, respectively (Figure 3g). In addition, H1 was largely found in Southeast Asian countries, while H4 was mainly found in Southern Europe and East Asia (Figure 3h). Taken together, these results suggested that the *OsbZIP08* confers the *indica*‐*japonica* differentiation with different geographical distribution in rice.

To investigate whether two major haplotypes (H1 and H4) have function difference, we constructed a genetic complementation transformation by introducing the allele of H1 from the *indica* variety 9311 into the H4 representative *japonica* variety ZH11. Three positive lines significantly increased plant height compared with wild type, but grain length, panicle length, and tiller number were identical to ZH11 (Figure 3i; Figure S6f,e), due to the stronger allele function with higher expression of *OsbZIP08* in ZH11. We also generated loss‐of‐function lines with frameshift mutations using CRISPR in ZH11 (Figure 3j). The grain length of three independent *osbzip08* mutants greatly increased (Figure 3j), consistent with the trend of the natural variation analysis (Figure 3e,f), but the plant height, tiller number, and panicle length of *osbzip08* mutants had no change (Figure 3j; Figure S6h). Thus, the results confirmed that natural variations of *OsbZIP08* negatively control grain size variation in rice.

### 2.4 OsGRX8 Redox‐modifies OsbZIP47 but Not OsbZIP08

**FIGURE 4:**
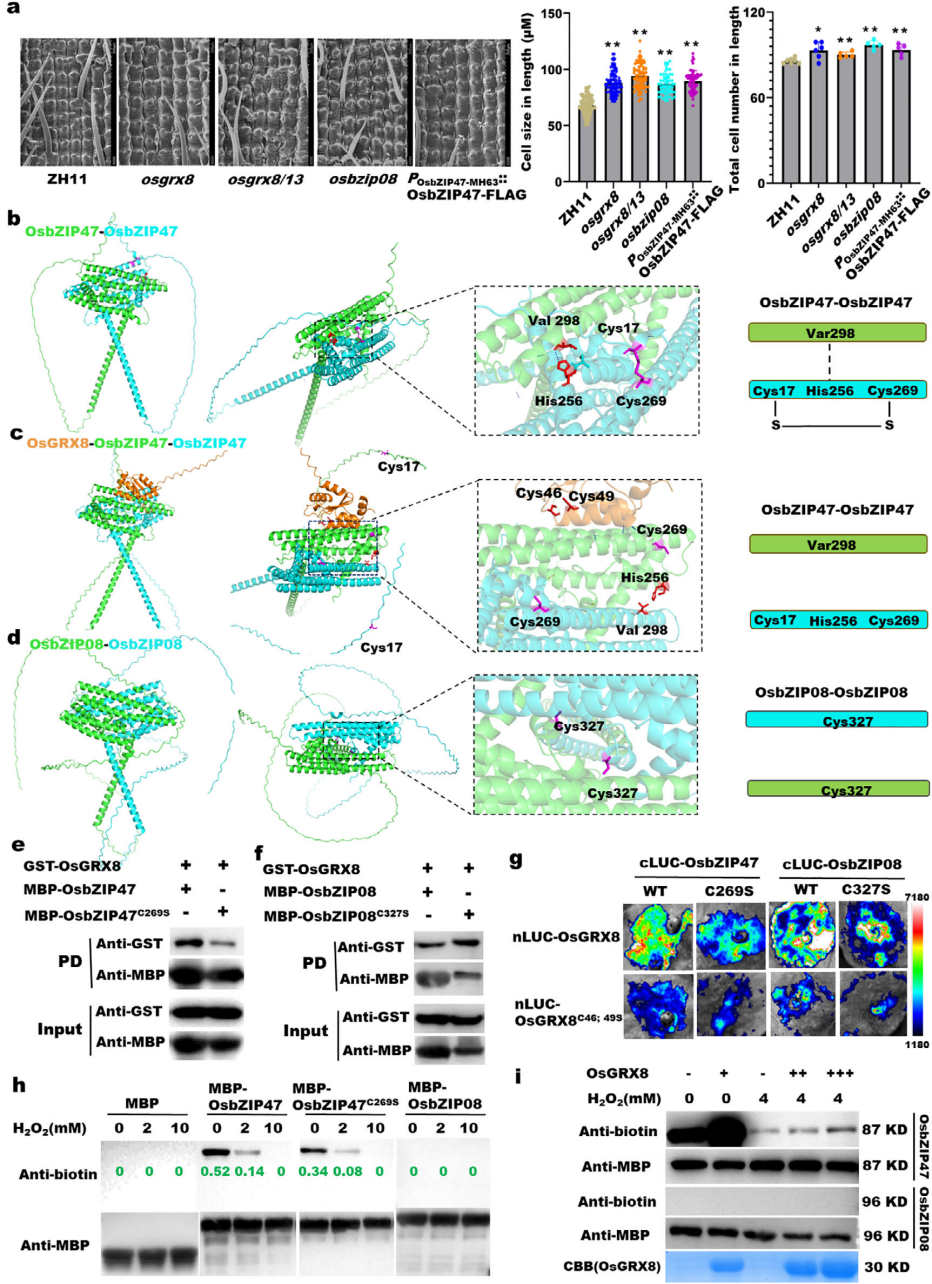
OsbZIP47 but not OsbZIP08 was redox‐modified by OsGRX8. (a) Cell length and cell number of mature hulls of various transgenic lines in the longitudinal direction by scanning electron microscope (SEM). All data are shown as mean value ± SEM. All the *p* values were produced by two‐tailed *t*‐tests. Asterisks indicated significant differences by Student's *t*‐test: ^*^ *p* < 0.05; ^**^ *p* < 0.01. (b–d) Structure predictions of OsbZIP47 homodimer (b), OsGRX8‐OsbZIP47 complex (c), and OsbZIP08 homodimer (d) using AalphaFold3 and visualization PyMOL software. Intramolecular disulfide bond was marked in a solid line, and intermolecular force was showed using a dotted line. (e) Pull‐down assays for the interactions of OsGRX8 with OsbZIP47 and OsbZIP47^C269S^. (f) Pull‐down assays for the interactions of OsGRX8 with OsbZIP08 and OsbZIP08^C327S^. (g) Split‐LUC assays for the interactions of OsGRX8, OsGRX8^C46S; 49S^ with OsbZIP47, OsbZIP08, and their mutations, respectively. (h) The redox modification of OsbZIP47, OsbZIP47^C269S^, and OsbZIP08 oxidized by H_2_O_2_ using the BIAM‐labelling assay. BIAM can label the reducing thiol group of a protein, so the amount of BIAM labeling represented the reduction degree of protein. Purified MBP protein was used as control. Quantification using Image J was marked in green. (i) The redox state of OsbZIP47 and OsbZIP08 modified by OsGRX8 using BIAM‐labelling assays.

To determine the cytological effect of *OsbZIP47*, *OsbZIP08*, and *OsGRX8*, we observed the mature hulls of ZH11, *osgrx8*, *osgrx8/13*, *osbzip08*, and *P_OsbZIP47_*_‐9311_::*OsbZIP47*‐FLAG complementation lines using scanning electron microscope. The results showed that cell size and total number in length of *osgrx8*, *osgrx8/13*, *osbzip08*, and *P_OsbZIP47_*_‐9311_::*OsbZIP47*‐FLAG were higher than wild type ZH11, indicating that *OsbZIP47*, *OsbZIP08*, and *OsGRX8* function in cell expansion and division (Figure 4a). The overlapping expression patterns of *OsbZIP08*, *OsbZIP47*, and *OsGRX8* in young panicles (Figure S7), as well as their similar functions on grain length and cellular effects indicate that they might have some relationship in grain size control in rice.

To further assess the molecular function of *OsGRX8*, *OsbZIP47*, and *OsbZIP08*, we conducted phylogenetic tree and domain analysis with their homologs found in different plant species. We identified that OsGRX8 has high identify with MSCA1 in maize and ROXY1 in *Arabidopsis* (Figure S8a), while OsbZIP47 and OsbZIP08 possess the basic region leucin zipper (BRLZ) and DOG1 domains and share significant similarity with ZmFEA4 and AtPAN (Figure S8b). It is reported that GRX utilizes its active cysteine site in the CCMC domain attacking the disulfide bond of target proteins [18]. Protein sequence alignment showed that there are two extremely conserved cysteine sites of Cys46 and Cys49 in the conserved CCMC domain of OsGRX8 (Figure S9a), and the extremely conserved cysteine site (Cys269) and two unique cysteine sites (Cys17 and Cys197) in OsbZIP47, as well as the only non‐conserved cysteine site (Cys327) in OsbZIP08 (Figure S9b). Taken together, these results indicated that Cys46 and Cys49 of OsGRX8, Cys17 and Cys269 of OsbZIP47, and Cys327 of OsbZIP08 may play notable roles for their functions.

To decipher the molecular mechanism underlying how these genes regulate grain length, we first predicted their protein structure using AalphaFold3. The results showed that OsbZIP47 or OsbZIP08 homodimer exhibited the typical structure of bZIP transcription factors (Figure 4b–d). There are some acting forces such as an intramolecular disulfide bond between Cys17 and Cys269 in OsbZIP47 and an intermolecular force between Val298 and His256 (Figure 4b). Once a mutation at the Cys17 and Cys269 of OsbZIP47, the damaged disulfide bond led to the conformation change, which inhibited the formation of OsbZIP47 homodimer (Figure S10a,b). Notably, OsGRX8 not only disturbs the intramolecular disulfide bond between Cys17 and Cys269 of OsbZIP47, but also breaks the intermolecular force between Val298 and His256 (Figure 4c), which is corresponding to the results of mutation at the Cys17 and Cys269 of OsbZIP47 (Figure S10c). While OsbZIP08 homodimer does not possess a disulfide bond at Cys327 (Figure 4d), and there is no change either mutating the Cys327 of OsbZIP08 or adding OsGRX8 (Figure S10d,e). These results suggested that OsGRX8 may modify the Cys17‐Cys269 intramolecular disulfide in OsbZIP47 homodimer but not OsbZIP08 homodimer.

Additionally, pull‐down, split‐LUC, and Y2H experiments and verified the physical interactions of OsGRX8 with OsbZIP47 and OsbZIP08 in vivo and in vitro (Figure 4e–g; Figure S11a). To further decipher the interaction specificity, the mutations of Cys46 and Cys49 of OsGRX8 (OsGRX8^C46S; C49S^), Cys269 of OsbZIP47 (OsbZIP47^C269S^) and Cys327 of OsbZIP08 (OsbZIP08^C327S^) were introduced. The physical interaction between OsGRX8 and OsbZIP47 was reduced when Cys269 of OsbZIP47 was mutated (Figure 4e), suggesting that Cys269 of OsbZIP47 is vital for its interaction with OsGRX8. The interaction and structure remain unchanged despite the Cys327 of OsbZIP08 was mutated (Figure 4f; Figure S11b), suggesting that the Cys327 of OsbZIP08 is not so important for its interaction with OsGRX8. Furthermore, split‐LUC and Y2H assays showed that OsGRX8^C46S; C49S^ can effectively weaken its interaction with OsbZIP08 or OsbZIP47 (Figure 4g; Figure S11a). The reason might be that although OsGRX8^C46S; C49S^ could not break the intramolecular disulfide bond between Cys17‐Cys269 of OsbZIP47, it completely destroyed the intermolecular force between Val298 and His256 (Figure S11c). The interaction specificity above indicated that the Cys46 and Cys49 of OsGRX8 and the Cys269 of OsbZIP47 are essential for their interactions, which directly contributes to redox reactions.

To assess whether OsbZIP47 and OsbZIP08 that contain redox‐sensitive cysteine residues can be oxidized by H_2_O_2_ and reduced by the OsGRX8 protein, we performed a biotin conjugated iodoacetamide (BIAM) labelling assay that is applied to test the redox sensitivity of a protein [41], and the flowchart is showed (Figure S11d). We found that OsbZIP47 was labelled by BIAM, and the labelled strength was dramatically decreased with an increased amount of H_2_O_2_, and the labeled OsbZIP47^C269S^ was obviously reduced even without H_2_O_2_, but OsbZIP08 was not labelled by BIAM (Figure 4h). Additionally, the reduction state of OsbZIP47 but not OsbZIP08 significantly increased by adding the OsGRX8 protein, and the increase was greatly weakened by high concentration H_2_O_2_ (Figure 4i). These findings confirmed that the redox status of OsbZIP47 but not OsbZIP08 is modified by OsGRX8 and the Cys269 of OsbZIP47 is important for the H_2_O_2_‐sensitive redox modification.

### 2.5 *OsbZIP47* Interacts with *OsbZIP08* Biochemically and Genetically

**FIGURE 5:**
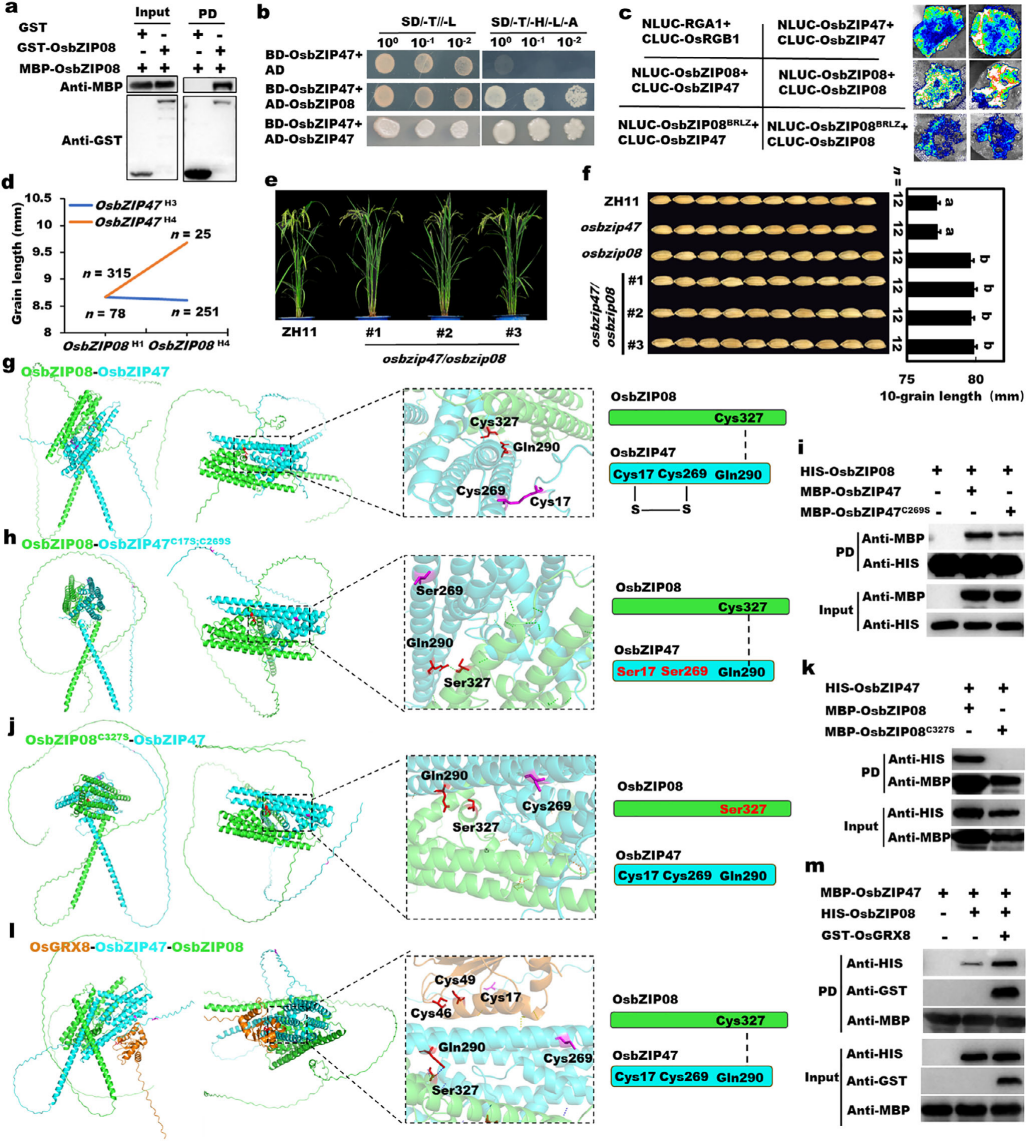
*OsbZIP47* interacts with *OsbZIP08* biochemically and genetically. (a) Pull‐down assay for the interaction of OsbZIP08 and OsbZIP08. (b) Y2H assays for the interactions of OsbZIP47 with OsbZIP47 and OsbZIP08 in vitro. AD, GAL4 activation domain; BD, GAL4 DNA‐binding domain. (c) Split‐LUC assays checked the interactions of OsbZIP47 and OsbZIP08 with OsbZIP08 and OsbZIP08^BRLZ^, respectively. c Pull‐down assays for the interactions of OsbZIP47 with OsbZIP47 or OsbZIP47^C269S^. (d) Grain length of four genotypes of *OsbZIP47* and *OsbZIP08* in 2013 accessions. (e) Plant architecture of three independent double mutants in T_2_. (f) Grain length of the *osbzip47/osbzip08* double mutants compared to *osbzip08, osbzip47*, and ZH11 in T_2_. *n* is the number of accessions of each haplotype or individuals of each transgenic line. All data are shown as mean value ± SEM. All the *p* values were produced by two‐tailed *t*‐tests. (g,h) Structure prediction of the OsbZIP47‐OsbZIP08 (g) and OsbZIP08‐OsbZIP47^C17S; C269S^ heterodimers (h). (i) Pull‐down assays for the interactions of OsbZIP08 with OsbZIP47 or OsbZIP47^C269S^. (j) Structure prediction of the OsbZIP47‐OsbZIP08^C327S^ heterodimer. (k) Pull‐down assays for the interactions of OsbZIP47 with OsbZIP08 or OsbZIP08^C327S^. (l) Structure prediction of the OsGRX8‐OsbZIP47‐OsbZIP08 complex. (m) The interactions of OsbZIP08 with OsbZIP47 or OsbZIP47^C269S^ were enhanced by adding the OsGRX8 protein via pull‐down assays.

To uncover the molecular mechanism between the two transcription factors of OsbZIP47 and OsbZIP08, we conducted Y2H, split‐LUC, and pull down assays. The results showed that OsbZIP47 and OsbZIP08 form homodimer or heterodimer in vivo and in vitro, which is corresponding to the results of structure predictions (Figure 5a–c; Figure S12a–c). In addition, there are interaction forces among the helixes in the DOG1 domain of C terminal and in the BRLZ domain of N terminal in OsbZIP47 or OsbZIP08, and OsbZIP47 or OsbZIP08 interacts with the same domain of OsbZIP08 (Figure 5c; and Figure S12a–e), suggesting that OsbZIP47 may competitively combine OsbZIP08 to inhibit the formation of OsbZIP08 dimer.

To explore the genetic relationship of *OsbZIP47* and *OsbZIP08*, we first analyzed the grain length phenotypes of the four genotypes determined by the two major haplotypes of each gene in 2013 accessions (Figure 5d; Table S6). We observed that the grain‐length difference (1.07 mm) between accessions carrying the two genotypes of *OsbZIP08* ^H4^–*OsbZIP47* ^H4^ and *OsbZIP08* ^H4^–*OsbZIP47* ^H3^ was much larger than that (0 mm) between accessions with the genotypes of *OsbZIP08* ^H1^–*OsbZIP47* ^H4^ and *OsbZIP08* ^H1^–*OsbZIP47* ^H3^ (Figure 5d). These results suggested that *OsbZIP47* has a genetic interacting effect over *OsbZIP08* in regulating grain‐length variation. Moreover, we generated the double mutants of *OsbZIP08* and *OsbZIP47* by CRISPR (Figure S12f). The plant and panicle morphologies of the positive *osbzip47*/*osbzip08* lines were similar to wild type (Figure 5e; Figure S12g), but the grain length of the double mutants of *osbzip47*/*osbzip08* were similar with that of the single mutant *osbzip08*, which exhibited longer grains than the *osbzip47* and wild type (Figure 5f). These results indicate that *OsbZIP08* genetically acts downstream of *OsbZIP47*.

To further decipher the interaction specificity, we introduced the mutations in the key sites. OsbZIP08 and OsbZIP47 can compose the typical and integral structure of bZIP transcription factor, and there exit intramolecular disulfide bond of Cys17‐Cys269 and intermolecular force of Gln290‐Cys327 in OsbZIP47‐OsbZIP08 heterodimer (Figure 5g). The interactions of OsbZIP47^C269S^ with OsbZIP47 or OsbZIP08 was evidently attenuated compared with wild type, despite of the intermolecular force of Gln290‐Cys327 is exiting, referring that the Cys17‐Cys269 disulfide bond affects the OsbZIP47‐OsbZIP08 interaction but not Gln290‐Cys327 intermolecular force (Figure 5h,i). Moreover, the Cys327 site of OsbZIP08 could completely abolish the interaction of OsbZIP47 and OsbZIP08 (Figure 5j,k). In addition, OsGRX8 enhanced the interaction of OsbZIP08 and OsbZIP47 probably by breaking the Cys17‐Cys269 disulfide bond of OsbZIP47 (Figure 5l,m). These results suggested that the two transcription factors OsbZIP08 and OsbZIP47 can interact with each other to form heterodimers in vivo and *in* *vitro*, and the C269 of OsbZIP47 and C327 of OsbZIP08 are essential for the interaction.

### 2.6 Self‐expression of *OsGRX8* Through the OsGRX8‐(OsbZIP47)‐OsbZIP08 Modules for Grain Size Homeostasis

To further explore the molecular mechanism on *OsGRX8*, *OsbZIP47*, and *OsbZIP08*, we subtly found that the variation vg0218413503 leads to a variation of a *cis*‐element of bZIP transcription factors between two subspecies: TGACG motif in *indica* varieties (MH63, 9311, ZS97) and TGACA motif in *japonica* varieties (NIP, ZH11) (Figure S13a,b).

**FIGURE 6:**
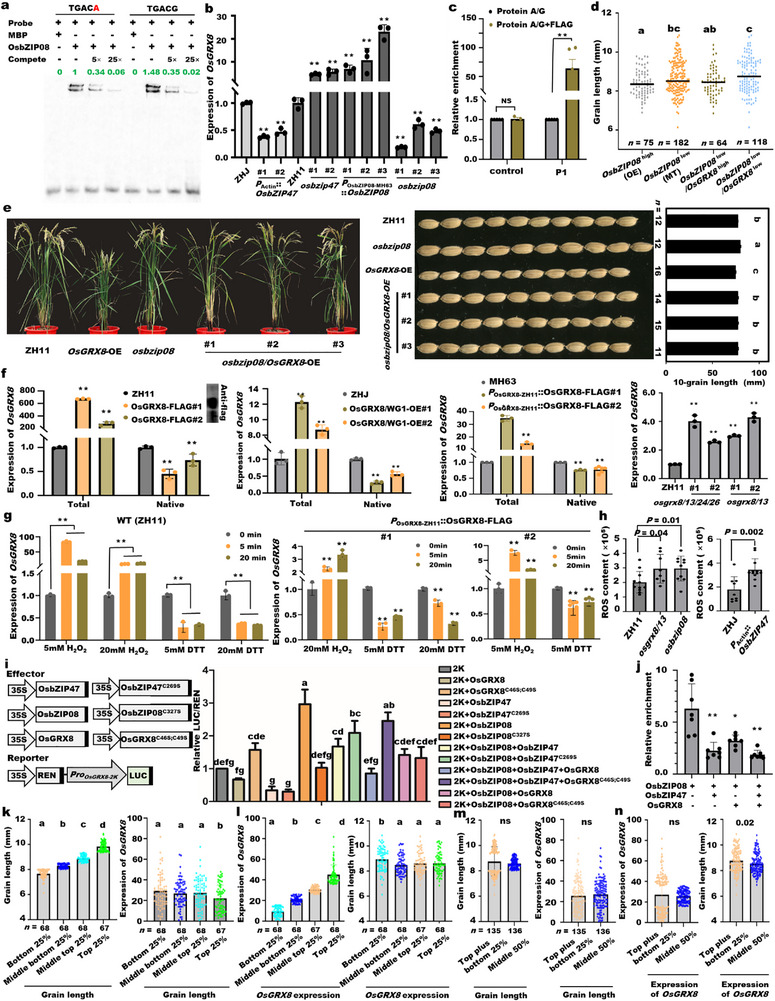
The self‐expression regulation of *OsGRX8* through the OsGRX8‐(OsbZIP47)‐OsbZIP08‐mediated negative feedback loops for grain size homeostasis. (a) Binding difference of OsbZIP08 on TGACG and TGACA motif using the EMSA assay. The binding extent was quantified using image J. (b) Expression levels of *OsGRX8* in 4‐cm panicles of WT and *P*_Actin_::*OsbZIP47* lines in the background of ZHJ, and *osbzip47*, *osbzip08* mutants, and *P_OsbZIP08_*_‐9311_::*OsbZIP08*‐FLAG plants in the background of ZH11. (c) ChIP‐qPCR assays for the *OsGRX8* enrichment on the targets using 4‐cm panicles of *P_OsbZIP08_*_‐9311_::*OsbZIP08*‐FLAG plants. Protein A/G as the negative control. The P1 at the bZIP binding site is the specific primer for ChIP‐qPCR. (d) Grain length of the different genotypes with the differential expression of *OsbZIP08* and *OsGRX8* in 257 accessions. Accessions with high or low expression of *OsbZIP08* are considered as overexpression (OE) or mutant (MT), respectively. (e) Plant architecture and grain length of WT (ZH11), *osbzip08*, *OsGRX8*‐OE, and *osbzip08/OsGRX8*‐OE lines. (f) The total and native expression levels of *OsGRX8* in 4‐cm panicles of *P*_Ubi_::*OsGRX8*‐FLAG, *P*_Actin_::*OsGRX8*/WG1‐OE, *P*_*OsGRX8*‐ZH11_::*OsGRX8*‐FLAG, *osgrx8/13*, and *osgrx8/13/24/26* lines were determined by qRT‐PCR using the specific primers marked in Figure S15d. #1 and #2 represent independent transgenic lines. Western blot assay checked the OsGRX8 in 4‐cm panicles of ZH11 and OE lines using FLAG antibody. (g) The native expression of *OsGRX8* in 4‐cm panicles of ZH11 and *P*_*OsGRX8*‐ZH11_::*OsGRX8*‐FLAG lines treated by H_2_O_2_ or DTT for 5 min or 20 min, separately. (h) Quantitative analysis of ROS contents in panicles of *osgrx8/13*, *osbzip08*, and *P*_Actin_::*OsbZIP47* lines determined by fluorescent probe DCFH‐DA. (i) Dual‐luciferase assays checked the activity of *OsGRX8* promoter regulated by various combinations of OsbZIP08, OsbZIP47 and OsGRX8 in *N. benthamiana* leaves. (j) ChIP‐qPCR assays for the binding activity of OsbZIP08 on the promoter of *OsGRX8* regulated by OsbZIP47 or OsGRX8 using 4‐cm panicles of *P_OsbZIP08_*_‐9311_::*OsbZIP08*‐FLAG, *P*_Ubi_::*OsbZIP47* and *P*_Ubi_::*OsGRX8* plants. (k) Grain length and *OsGRX8* expression among accessions quartered by grain length in 271 accessions. (l) *OsGRX8* expression and grain length among accessions quartered by *OsGRX8* expression level in 271 accessions. (m) Grain length and *OsGRX8* expression between the top plus bottom 25% and middle 50% of grain length in 271 accessions. (n) *OsGRX8* expression and grain length between the top plus bottom 25% and middle 50% of *OsGRX8* expression level in 271 accessions. All the *p* values were produced by the two‐tailed *t*‐tests (^*^, *p* < 0.05; ^**^, *p* < 0.01, ns represents no significance). Significant differences indicated by different letters via one‐way ANOVA and Duncan's test. Means ± SEM.

To investigate whether OsbZIP47 and OsbZIP08 have binding difference on the two motifs, we conducted electrophoretic mobility shift assay (EMSA) by synthetizing their probes and found that OsbZIP08 bound TGACG probe more obviously than that of TGACA probe (Figure 6a). But there was no difference on the binding ability of OsbZIP47 on the two motifs (Figure S13c). Furthermore, we discovered that the binding ability of OsbZIP08 on the *OsGRX8* promoter was stronger than that of OsbZIP47 (Figure S13d), which was significantly reduced at the presence of OsbZIP47, OsGRX8 or OsGRX8 and OsbZIP47 (Figure S13d,e).

To further examine the regulation of *OsGRX8* by *OsbZIP47* or *OsbZIP08*, we first analyzed the expression of *OsGRX8* between the relatively high and low expression accessions of *OsbZIP47* or *OsbZIP08* in the mini‐core collection with 271 accessions (Figure S14a,b; Table S8). The results showed that the expression level of *OsGRX8* in 136 accessions with relatively high expression of *OsbZIP47* was much lower than that in 132 accessions with low expression of *OsbZIP47*, which contributed to the longer grains (Figure S14a), indicating that *OsbZIP47* negatively regulates *OsGRX8* expression. Furthermore, the expression level of *OsGRX8* in the 149 accessions with relatively high expression of *OsbZIP08* was obviously higher than the 114 accessions with low expression of *OsbZIP08*, which lead to the shorter grains (Figure S14b), suggesting that *OsbZIP08* positively regulates *OsGRX8* expression. These results further indicate that *OsbZIP47* negatively and *OsbZIP08* positively regulate *OsGRX*8, which negatively regulates grain length variation in rice.

To confirm this hypothesis that *OsGRX8* is the downstream gene of both OsbZIP08 and OsbZIP47, the transcriptional changes of *OsGRX8* in mutant and OE transgenic plants of upstream factors were subsequently verified by Real‐time PCR (Figure 6b). It was showed that the *OsGRX8* expression was significantly inhibited in overexpression lines of *OsbZIP47*, but was greatly up‐regulated in the *osbzip47* mutant (Figure 6b), confirming that *OsGRX8* is negatively regulated by OsbZIP47. Meanwhile, *OsGRX8* transcripts were visibly reduced in *osbzip08* mutants and obviously induced in young panicles of *P_OsbZIP08_*_‐9311_::*OsbZIP08*‐FLAG plants (Figure 6b), verifying that *OsGRX8* is positively regulated by OsbZIP08. Furthermore, chromatin immunoprecipitation quantitative PCR (ChIP‐qPCR) analysis using young panicles of *P_OsbZIP08_*_‐9311_::*OsbZIP08*‐FLAG transgenic lines showed that OsbZIP08 bound the promoter of *OsGRX8 in vivo* (Figure 6c). All the findings indicated that *OsGRX8* expression was regulated by the OsbZIP47‐OsbZIP08 transcriptional complex.

To explore the genetic relationship supporting for *OsGRX8* with *OsbZIP47* and *OsbZIP08*, we conducted the analysis of grain length among four genotypes of *OsGRX8* with *OsbZIP47* and *OsbZIP08* in the rice min‐core collection of 2013 accessions, respectively (Table S3, S5, S7, and S9). These results suggested that *OsGRX8* has a genetic interacting effect over *OsbZIP08* and *OsbZIP47* in negatively regulating grain length variation (Figure S14c). To verify the genetic relationship between *OsbZIP08* and the downstream *OsGRX8* gene, we first analyzed the phenotype difference of grain length with different expression levels of the two genes in 257 accessions, and the results showed that the high expression of *OsGRX8* could recover the grain‐length phenotype of accessions with low *OsbZIP08* expression to the level of accessions with high *OsbZIP08* expression (Figure 6d). Furthermore, we obtained the genetic material of over‐expressing *OsGRX8* in *osbzip08* mutant background. The grain length of three *osbzip08/OsGRX8*‐OE lines recovered the grain size of *osbzip08* mutants to the wild‐type level, despite of no change in plant and panicle morphologies (Figure 6e; Figure S15a–c), which further confirmed that *OsGRX8* genetically and transcriptionally acts downstream of *OsbZIP08* for grain length control.

To confirm whether the endogenous expression of *OsGRX8* was regulated by OsGRX8 protein, we determined the total and native expression level of *OsGRX8* by qRT‐PCR using 4‐cm young panicles of *OsGRX8*‐related genetic materials (Figure S15d). The endogenous *OsGRX8* transcripts were obviously reduced in *OsGRX8* OE lines and *P*_*OsGRX8*‐ZH11_::*OsGRX8*‐FLAG complementation plants, and increased in *osgrx8/13* or *osgrx8/13/24/26* mutants (Figure 6f), confirming that the transcriptional level of *OsGRX8* was inhibited by its own protein.

It has been established that the oxidized state of a glutaredoxin was significantly increased by adding H_2_O_2_ and decreased by adding DTT [42, 43]. Accordingly, ZH11 (WT) and *P*_*OsGRX8*‐ZH11_::*OsGRX8*‐FLAG complementation plants were treated with different concentration of H_2_O_2_ or DTT. The native mRNA level of *OsGRX8* could be significantly increased by adding H_2_O_2_, and the results were opposite when treated with DTT (Figure 6g). Furthermore, ROS contents in panicles of *osgrx8/13*, *osbzip08*, and *P*_Actin_::*OsbZIP47* lines were observably increased (Figure 6h; Figure S16), suggesting that *OsGRX8* indeed changes redox status in vivo. All the data confirmed that the oxidized and reduced states of OsGRX8 enhanced and inhibited the self‐expression of *OsGRX8*, respectively.

To investigate the effects of the OsGRX8‐(OsbZIP47)‐OsbZIP08 modules on the expression of the *OsGRX8* gene, we performed a dual‐luciferase transactivation assay in *N. benthamiana* leaves (Figure 6i). *OsGRX8* expression was activated by OsbZIP08 alone but not by OsbZIP08^C327S^ (Figure 6i), showing an essential role of the Cys327 site of OsbZIP08 in the transcriptional activation of *OsGRX8*. The transcriptional activity of OsbZIP08 on *OsGRX8* was obviously inhibited by adding OsbZIP47, and the inhibition was further strengthened in the presence of OsGRX8 protein but not the OsGRX8^C46S; C49S^ protein (Figure 6i), showing a redox‐dependent transcriptional regulation by the OsGRX8‐OsbZIP47‐OsbZIP08 module. The transcriptional activity of OsbZIP08 on *OsGRX8* was obviously inhibited by adding either OsGRX8 or OsGRX8^C46S; C49S^ protein (Figure 6i), showing a redox‐independent transcriptional regulation by the OsGRX8‐OsbZIP08 module. Furthermore, ChIP‐qPCR analysis suggested that the binding activities of OsbZIP08 on *OsGRX8* were inhibited by adding either OsbZIP47 or OsGRX8 or both of them (Figure 6j). These results convincingly confirmed that the expression of *OsGRX8* is fine‐tuned by the OsGRX8‐(OsbZIP47)‐OsbZIP08 modules and their redox states.

To further explore the relationship of grain length and the expression of *OsGRX8* in a natural mini‐core collection, we found grain length varied with the expression level of *OsGRX8*, and their correlation coefficient was ‐0.38 in 31 *japonica* accessions (Figure S17a; Table S9). Furthermore, the *OsGRX8* expression pattern among accessions quartered by grain length (Figure 6k) was highly similar with that of grain length quartered by *OsGRX8* expression (Figure 6l), showing a high correlation between grain length and *OsGRX8* expression level in the rice mini‐core collection. When there is a grain length homeostasis between the top plus bottom 25% and middle 50% of grain length in the natural population, there exists an *OsGRX8* expression homeostasis between them (Figure 6m). Furthermore, when there is an *OsGRX8* expression homeostasis between the top plus bottom 25% and middle 50% of *OsGRX8* expression level in the natural population, there is a grain length homeostasis between them (Figure 6n). These results further suggest that the fine‐tune regulation of *OsGRX8* expression generally controls grain size homeostasis in the rice natural population.

Collectively, the results confirmed that OsGRX8 negatively regulates the self‐expression of *OsGRX8* by weakening the binding of OsbZIP08 on its promoter through enhancing its interaction with both OsbZIP47 and OsbZIP08. The OsGRX8‐(OsbZIP47)‐OsbZIP08 modules employ redox‐dependent and ‐independent pathways to regulate the self‐expression of *OsGRX8*, which forms a negative feedback mechanism to maintain grain size homeostasis among rice subspecies.

### 2.7 Self‐Regulatory Haplotypes (SRHs) Exhibit *Indica*‐*Japonica* Differentiation and Greatly Contribute to Yield Traits in Rice

**FIGURE 7:**
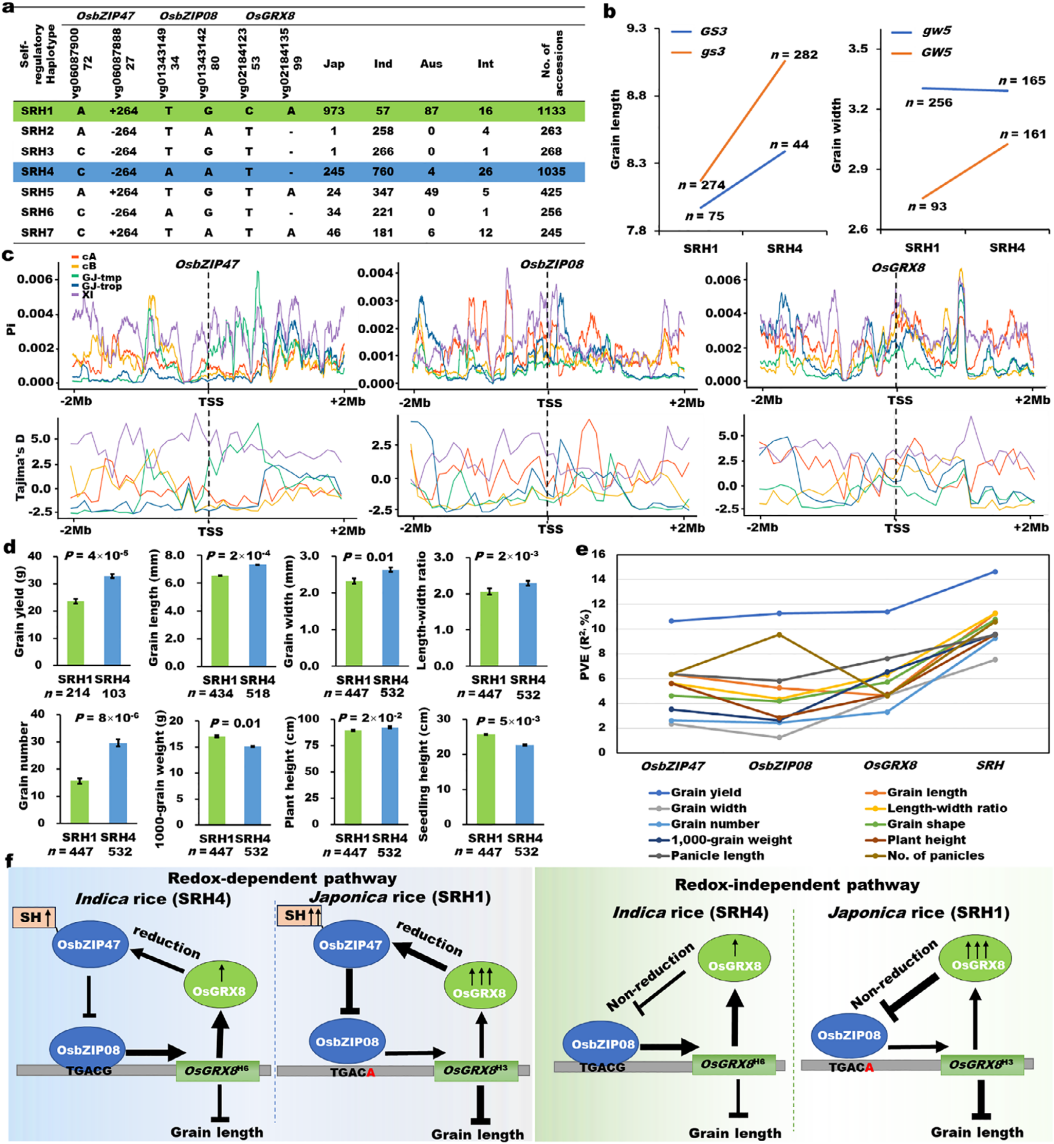
Self‐regulatory haplotypes (SRHs) for *indica*‐*japonica* differentiation and yield contribution and a regulatory model for grain size homeostasis by the OsGRX8‐(OsbZIP47)‐OsbZIP08‐*OsGRX8* loops in rice. (a) SRHs caused by co‐selected natural variations of the three genes in 4726 accessions and the two haplotypes (SRH1 and SRH4) confer to *indica*‐*japonica* differentiation. (b) Genetic interactions between SRHs and two major grain‐size QTL *GS3* or *GW5* using their functional variations. (c) Selective sweep analyses (Pi and Tajima's D) for *OsbZIP47*, *OsbZIP08*, and *OsGRX8* in 3000 accessions. cA, cB, GJ‐tmp, GJ‐trop, XI represent Aus, Basmati, Temperate *japonica*, Tropical *japonica*, and *Indica* accessions, respectively. (d) Grain yield traits between SRH1 and SRH4 in 4726 accessions. (e) Genetic contributions of the three genes and SRHs to grain yield traits in 4726 accessions. The phenotype variance explanation (PVE), which was determined by the one‐way or multiple ANOVA using the haplotypes of each gene and SRHs as the predictor variables and the yield traits of 533 accessions, was used to assess the genetic contribution of each gene and SRH. (f) A regulatory model for grain size homeostasis and *indica*‐*japonica* differentiation by the OsGRX8‐(OsbZIP47)‐OsbZIP08‐*OsGRX8* loops in redox‐dependent or ‐independent manners. In the redox‐dependent pathway, OsGRX8 controls the posttranslational reduction modification of OsbZIP47 thereby increasing OsbZIP47‐OsbZIP08 interaction to further inhibit the transcriptional activity of OsbZIP08 on *OsGRX8*. While in the redox‐independent pathway, OsGRX8 directly interacts with OsbZIP08 independent of the reduction reaction to inhibit its transcriptional activation on *OsGRX8*. Furthermore, natural variations in the OsGRX8‐(OsbZIP47)‐OsbZIP08‐*OsGRX8* modules confer *indica*‐*japonica* differentiation. *Indica* rice with long and thin grains, OsbZIP08 binds tightly on TGACG motif of *OsGRX8* ^H6^ to promote its expression level. Furthermore, the low OsGRX8 level weakly inhibits the transcription activation and binding activity of OsbZIP08 on *OsGRX8* ^H6^, which is directly regulated in a redox‐independent way and indirectly by moderating the reducing power of OsbZIP47 in a redox‐dependent way, leading to the induced expression of *OsGRX8* ^H6^. While in *japonica* rice with short and wide grains, OsbZIP08 binds slightly on TGACA motif of *OsGRX8* ^H3^ to weakly promote its expression level. Furthermore, high OsGRX8 significantly inhibits the transcriptional activation and binding activity of OsbZIP08 on *OsGRX8* ^H3^ by the redox‐independent way and redox‐dependent way that increases the reducing power of OsbZIP47, leading to a bit expression of *OsGRX8* ^H3^. Single, two, or three arrows represent low or high expression level and modification extent, respectively. *n* is the number of accessions of each haplotype. All data are shown as mean value ± SEM. All the *p* values were produced by the two‐tailed *t*‐tests.

We analyzed the representative variations of *OsbZIP47*, *OsbZIP08*, and *OsGRX8*, and surprisingly found that most of their genotypes co‐segregated or co‐selected with each other in 4726 rice accessions (Table S10), although they were located on three different chromosomes. We thus combined these three genes together for further haplotype analysis and identified seven self‐regulatory haplotypes (SRHs), caused by the co‐selected variations of the three genetically unlinked genes that formed the negative feedback loops, in the 4726 accessions (Figure 7a). SRH1 and SRH4 were the two major SRHs, accounting for the majority of 4726 accessions, predominant in *japonica* (85.9%) and *indica* (73.4%) subspecies, respectively (Figure 7a; Figure S17b). In addition, to determine the position of SRHs in the existing grain size regulatory networks, we analyzed the genetic interaction between SRHs and two major grain size QTL *GS3* and *GW5* using their function variations (Figure 7b). The results showed that the grain‐length difference (0.2 mm) between accessions carrying the two genotypes of SRH1‐*GS3* and SRH1‐*gs3* was much smaller than that (0.67 mm) between accessions with the genotypes of SRH4‐*GS3* and SRH4‐*gs3* (Figure 7b). Similarly, the grain‐width difference (0.54 mm) between accessions carrying the two genotypes of SRH1‐*gw5* and SRH1‐*GW5* was much larger than that (0.27 mm) between accessions with the genotypes of SRH4‐*gw5* and SRH4‐*GW5* (Figure 7b). These results suggested that there are genetic interactions between SRHs and *GS3* and *GW5*, which inferred that they corporate with the major grain‐size regulatory networks.

Furthermore, we performed Pi and Tajima's D analysis using a natural population of 3000 accessions. Extremely high positive D and modest Pi values in XI/*indica* suggested that *OsbZIP47* experienced balanced selection, while low Pi and negative D values in cB and GJ‐trop/*japonica* suggested that *OsbZIP47* experienced strong positive selection (Figure 7c). In additions, *OsbZIP08* and *OsGRX8* may undergo balanced selection in XI/*indica* and positive selection in cB, GJ‐tmp/*japonica*, and GJ‐trop/*japonica* with remarkably low Pi values (Figure 7c). These results indicate *OsbZIP47*, *OsbZIP08*, *OsGRX8*, and their SRHs show high genetic differentiation between *indica* and *japonica* subspecies. Together, these observations suggest that *OsbZIP47*, *OsbZIP08*, and *OsGRX8* represent critical adaptive targets under strong selection.

To explore the functional difference of SRHs, we analyzed multiple yield traits between the two major SRHs (SRH1 and SRH4) (Figure 7d). SRH4 has enhanced grain yield, grain length, grain width, grain length‐width ratio, and grain number compared to SRH1 (Figure 7d). These results were highly consistent with the typical differentiation traits between *indica* and *japonica* rice, and further indicated that the two major SRHs have important effects on the inter‐subspecies differentiation and grain yield traits in rice.

Using the representative variant genotypes of these three genes and the phenotypes of 4726 accessions, ANOVA was performed to evaluate the genetic contributions of these three genes and SRHs to the major yield traits (Figure 7e). *OsbZIP47*, *OsbZIP08*, *OsGRX8* and the two major SRH1 and SRH4 explained 10.7%–14.6% of grain yield variation, 4.6%–11.3% of grain length variation, 1.3%–7.5% of grain width variation, 4.4%–11.3% length‐width ratio variation, 2.5%–9.3% of grain number variation, 4.2%–10.8% of grain shape variation, 2.7%–9.6% of 1000‐grain weight variation, 2.9%–9.6% plant height variation, 5.8%–9.5% of panicle length variation, and 4.6%–10.6% of number of panicles variation in 4726 accessions, suggesting that the genetic contribution of SRHs to these yield traits was much higher than each single gene (Figure 7e). In addition, we analyzed the significance of epistatic effects among *OsbZIP47*, *OsbZIP08*, *OsGRX8*. The results suggested that the combination of *OsGRX8* and *OsbZIP47* had obviously synergistic/epistatic effects (Sig < 0.05) on grain length, while the combination of *OsbZIP47* and *OsbZIP08* had a similar effect on 1000‐grain weight (Sig < 0.05) (Figure S17c). But the other combinations among *OsbZIP47*, *OsbZIP08*, and *OsGRX8* showed simple additive effects (Sig > 0.05) on yield traits (Figure S17c). These results indicated that the SRHs of the three genes have a synergistic effect on the variation of yield traits in rice, and showed strong *indica*‐*japonica* differentiation and artificial selection signatures.

## 3 Discussion

Modeling the genetic architecture between genes and traits is a challenging endeavor, because complicated agronomic traits are cooperatively mediated by numerous QTGs [44]. A large number of QTGs controlling grain shape has been cloned, and the genetic interactions of QTGs has been reported [12, 44], however, the interaction mechanism among them to maintain a final grain size homeostasis remains poorly understood [12]. Herein, we discovered that natural variations in *OsGRX8*, *OsbZIP47*, and *OsbZIP08* identified by GWAS contribute significantly to *indica*‐*japonica* differentiation for grain size variation in rice (Figures 1, 2, 3). We for the first time confirmed the interactions among the three QTGs biochemically and genetically, which collaboratively regulate grain size homeostasis by controlling the self‐expression of *OsGRX8* using a negative feedback mechanism formed by them (Figures 4, 5, 6). We confirmed that natural negative feedback loops of two pathways for the OsGRX8‐(OsbZIP47)‐OsbZIP08 modules regulate the self‐expression of *OsGRX8* for grain size homeostasis between rice subspecies (Figure 7f). In brief, these findings confirmed that the OsGRX8‐OsbZIP47‐OsbZIP08‐*OsGRX8* and OsGRX8‐OsbZIP08‐*OsGRX8* negative feedback loops encoded by three interacted QTGs regulates the self‐expression of *OsGRX8* in redox‐dependent or ‐independent manners to ensure the stable expression of the glutaredoxin gene/protein for the stable redox state and to make the final grain size not too large or too small in rice, revealing a homeostasis regulation mechanism linking both redox and final organ size in crops. Furthermore, based on natural variations of the three genes conferring significant *indica*‐*japonica* differentiation, artificial selection and co‐selection signatures, we proposed a new concept, SRHs, haplotype combinations from co‐selected variations of the genetically unlinked genes with epistasis effects in a module, to form natural negative feedback loops for grain size homeostasis in rice. In *indica* rice with long and thin grains, low *OsGRX8* ^H6^ level weakly inhibits the transcription activation and binding activity of *OsbZIP08* ^H1^ on *OsGRX8* ^H6^, which leads to the induced expression of *OsGRX8* ^H6^, while the regulation in *japonica* rice with short and wide grains are opposite (Figure 7f). How two loops of OsGRX8‐OsbZIP47‐OsbZIP08‐*OsGRX8* and OsGRX8‐OsbZIP08‐*OsGRX8* synergistically regulate grain size homeostasis and *indica*‐*japonica* differentiation remains to be further researched.

In this study, both of *OsbZIP08* and *OsbZIP47* encode bZIP transcription factors, however, there are significant differences in protein biochemical activity and genetic function between them. First, OsbZIP08 and OsbZIP47 have function differences in grain length regulation: *OsbZIP47* positively regulates grain length (Figure 2i–j; Figure S5), while *OsbZIP08* possesses a negative function (Figure 3i,j). Furthermore, the OsbZIP47 protein but not OsbZIP08 could be modified by H_2_O_2_/OsGRX8 (Figure 4h,i), owning to the intramolecular disulfide bonds between Cys17 and Cys269 in OsbZIP47, while OsbZIP08 cannot form intermolecular disulfide bonds (Figure 4b,d). Additionally, OsbZIP08 positively and OsbZIP47 negatively regulate the expression of the downstream gene *OsGRX8* (Figure 6b,i). Besides, the binding ability of OsbZIP08 on the promoter of *OsGRX8* in *indica* is higher than that in *japonica*, leading the differential expression of *OsGRX8* between the two subspecies (Figure 6a), whereas OsbZIP47 binds indiscriminately on the two promoters (Figure S13c). OsbZIP08 has a stronger binding ability on the promoter of *OsGRX8* than that of OsbZIP47 (Figure S13d). Moreover, OsbZIP47 competitively combines OsbZIP08 to inhibit the formation of OsbZIP08 dimer, which inhibits the binding and transcriptional activation ability of OsbZIP08 on downstream genes of *OsGRX8*, revealing a new mechanism for the two important bZIP transcription factors. Collectively, OsbZIP47 and OsbZIP08 possess the totally opposite functions in a common module to achieve a synergistic and flexible homeostasis regulation, which is likely attributed to the significant difference in important cysteine sites. How the cysteine residues of OsbZIP47 and OsbZIP08 function for redox homeostasis and grain size homeostasis in vivo remains to be further studied.

Cellular redox status is essential to regulate basic biological processes including cell proliferation/differentiation, metabolic homeostasis, and stress responses, and GRXs plays an essential role in the control of the cellular redox states and related pathways in many organisms [20, 43]. CC type GRXs, specific oxidoreductases in plants, catalyze the reversible reduction of disulfide bonds of substrate proteins to regulate their activity [45]. It is reported that the functions of TGA/bZIP transcription factors were closely related to their redox‐dependent control, which emphasizes the importance of cysteine residues in bZIP proteins [27, 29, 46]. The underlying molecular mechanism for maintaining the redox homeostasis by GRXs is important but not clear in plants [26]. In this study, we found that OsGRX8 physically interacts with and directly participates in the post translational modification of OsbZIP47 by breaking the disulfide bonds between Cys17 and Cys269 in OsbZIP47, and the interaction is weakened by mutations in the conserved CCMC domain of OsGRX8 (OsGRX8^C46S; C49S^) or at Cys269 of OsbZIP47^C269S^ (Figure 4; Figures S10 and S11). Furthermore, mutation at the Cys269 of OsbZIP47 not only breaks the intramolecular disulfide bonds between Cys17 and Cys269 in OsbZIP47 but also disturbs intermolecular interaction forces in OsbZIP47‐OsbZIP08 heterodimer, despite the intermolecular interaction force of Cys327 and Gln290 is exiting (Figure 5h,i). The interaction between OsbZIP47 and OsbZIP08 was disappeared once the Cys327 of OsbZIP08 was mutated (Figure 5j,k). These results indicated that Cys269 of OsbZIP47 and Cys327 of OsbZIP08 play a vital role for the interaction between OsbZIP47 and OsbZIP08. Notably, the mutation in Cys269 of OsbZIP47 does not affect the function of OsbZIP47 in transcriptional repression on the downstream *OsGRX8* gene or in inhibiting the binding and transcriptional activation of OsbZIP08 on *OsGRX8* (Figure 6i). These features are consensus to TGA1^C260; C266^ in *Arabidopsis*: mutations at C260 and C266 sites inhibit the interaction ability between TGA1 and NPR1, but do not affect the binding and transcriptional activation characteristics of TGA1 [30, 47]. It is thus inferred that there are other cysteine residues in OsbZIP47 functioning in the pathway. In addition, OsGRX8 also increases the interaction between OsbZIP47 and OsbZIP08 to decrease the transcriptional activation activity of OsbZIP08 on *OsGRX8* (Figure 5i,m and Figure 6i,j; Figure S13d,e), which is inhibited by the mutations at the conserved CCMC domain of OsGRX8^C46S; C49S^ (Figure 6i). All the results indicated that the GRX‐bZIP module‐dependent redox reaction is highly conserved in plants and the OsGRX8‐OsbZIP47‐OsbZIP08‐*OsGRX8* feedback loop for grain size homeostasis depends on the redox homeostasis regulation, with conserved cysteines acting as a switch for the reactions.

Generally, GRXs for the post‐translational modification of proteins are dependent on redox reaction by attacking the disulfide bonds of the substrate proteins, but the molecular mechanism of GRXs that are independent on redox reaction is not clear. Here, we discovered the OsGRX8‐OsbZIP08‐*OsGRX8* regulatory module in that OsGRX8 directly interacts with OsbZIP08 and restrains the binding and the activation activity of OsbZIP08, leading to the decrease of *OsGRX8* expression (Figure 7f). First, OsbZIP08 does not possess a disulfide bond through the unique cysteine site Cys327 thus it cannot be reduced by OsGRX8 through redox reaction (Figure 4d,h). In addition, the mutation at Cys327 of OsbZIP08 makes no difference for the interaction between OsbZIP08 and OsGRX8 (Figure 4f). There results indicated that the interaction between OsGRX8 and OsbZIP08 is not dependent on disulfide bonds. Moreover, the activation activity of OsbZIP08^C327S^ on OsGRX8 was destroyed (Figure 6i), indicating that the Cys327 of OsbZIP08 plays a decisive role in its function. Notably, the interaction of OsGRX8^C46S; C49S^ with OsbZIP08 is weakened, but the transcriptional inhibiting activity of OsbZIP08 by OsGRX8^C46S; C49S^ or OsGRX8 is the same (Figure 4g and Figure 6i), which suggested that the conserved CCMC domain of OsGRX8 affects the interaction capacity of OsGRX8 with OsbZIP08, but has no effect on the transcriptional activity of OsbZIP08 on *OsGRX8*. Taken together, the CCMC domain of OsGRX8 plays an important role in the interaction between OsGRX8 and OsbZIP08 but it does not exert its redox modification function on OsbZIP08, and the Cys327 amino acid plays an important role in the transcriptional function of OsbZIP08 rather than the redox modification function of OsbZIP08. Moreover, OsGRX8 inhibits the transcription activation and the DNA binding ability of OsbZIP08 on *OsGRX8* probably not by affecting the forming of OsbZIP08 homodimer but changing the conformation of the basic DNA‐binding region in OsbZIP08 homodimer (Figure 4d and Figure 6i,j; Figures S10e and S18). Thus, the OsGRX8‐OsbZIP08‐*OsGRX8* negative feedback loop is independent of the redox reaction to sustain grain size homeostasis by fine‐tuning *OsGRX8* expression. How the OsGRX8 switch system senses its protein amount at a certain level range to ensure normal redox homeostasis for the maintenance of appropriate grain size remains to be investigated.

Herein, we identified that there exit two negative feedback loops of OsGRX8‐OsbZIP47‐OsbZIP08‐*OsGRX8* and OsGRX8‐OsbZIP08‐*OsGRX8* in redox‐dependent or ‐independent manners in regulating grain size genetically and biochemically. The functions of the two negative feedback loops are redundant in regulating the self‐expression of *OsGRX8*. The negative feedback loop OsGRX8‐OsbZIP47‐OsbZIP08‐*OsGRX8* is dependent on the appropriate redox environment *in* *vivo*. When the internal redox environment is beneficial, the loop could be initiated. Otherwise, the OsGRX8‐OsbZIP08‐*OsGRX8* module should be initiated to maintain the protein level of *OsGRX8*. This regulatory mechanism may be a bet‐hedging strategy evolved to adapt various environmental changes, which aims to make the final grain size not too large or too small and to maintain organ size homeostasis in rice.

Understanding the genetic architecture of major agronomic traits is vital to directly guide robust high yield rice breeding [48, 49, 50]. In this study, besides the insights into the complicated molecular 3mechanisms underlying grain size homeostasis, most strikingly, the natural variations of these genes contribute significantly to grain yield traits. The OsGRX8‐(OsbZIP47)‐OsbZIP08‐*OsGRX8* regulatory modules can be utilized for enhancing stable grain yield in breeding programs in cereals, due to their highly conserved proteins and the high heritability of grain size in crops. In additions, natural variations of the three genes confer to very strong *indica*‐*japonica* differentiation and artificial and co‐selection signatures, respectively. The most representative variations of the three genes were co‐selected in 4726 accessions, thus we renamed haplotype combinations from these genes and proposed the SRH concept to form a natural regulatory module with diverse forms and functions. Two major SRHs were identified to have a larger synergistic effect on variation of grain yield traits than each single gene and showed strong *indica*‐*japonica* differentiation and artificial selection signatures. Based on all the findings above, we attentively formulate the design that the combinations of the beneficial alleles of *OsbZIP47*, *OsbZIP08*, and *OsGRX8* may provide a new strategy for facilitating the development of high‐ and stable‐yield crop varieties. Thus, we would generate lines carrying various combinations of *OsbZIP47*, *OsbZIP08*, and *OsGRX8* alleles in elite varieties to obtain robust high yield rice cultivated in various environments.

## 4 Methods

### 4.1 Genome‐Wide Association Study (GWAS)

For the GWAS analysis [51], a sequenced diverse panel of the rice mini‐core collection containing 533 accessions was obtained and a standardized quality control (QC) pipeline was implemented on the genotype data using PLINK v1.9. Sequential filters were applied to exclude low‐quality samples and single‐nucleotide polymorphisms (SNPs). Samples with a missing genotype rate exceeding 10% and SNPs with a missingness rate above 5% were removed. And after filtering out some SNPs with a minor allele frequency (MAF) lower than 5%, the 4,131,700 SNPs with MAF > 5% were retained for analysis.

Following initial QC, linkage disequilibrium (LD) pruning was conducted to minimize spurious signals arising from correlated SNPs rather than true population structure. An iterative pruning approach was employed using the command “‐indep‐pairwise 50 10 0.2”, which slides a 50‐SNP window in 10‐SNP steps and removes one SNP from any pair with an r^2^ > 0.2. This process was repeated until no long‐range LD regions significantly influenced the PCA results, ensuring that the remaining SNPs captured genuine population stratification. The final high‐quality and LD‐pruned dataset was used for all subsequent population genetic analyses and GWAS.

The GWAS analysis was conducted by the factored spectrally transformed linear mixed model (FaST‐LMM) [52]. The Manhattan plots were mapped by the *qqman* package. Bonferroni correction was used to determine the threshold for genome‐wide significance (*p* = 0.05/*n* after correction, n is the effective number of independent SNPS in the whole genome). The genome‐wide significance level for grain size was determined as 1.37 × 10^−6^.

### 4.2 Sequence Variation and Haplotype Analysis

Sequence variations of *OsGRX8*, *OsbZIP47*, and *OsbZIP08* from 533 rice accessions were downloaded from the Rice Variation Map2.0 database (http://ricevarmap.ncpgr.cn) [40, 53]. Sequence variations in the 3‐Kb (*OsbZIP47* and *OsbZIP08*) and 4.5‐Kb (*OsGRX8*) promoter regions, the coding, and the 0.5‐Kb downstream regions were selected and used to perform haplotype analysis. The representative variations of rice accessions are listed in Tables S3, S5 and S8.

### 4.3 Field Growth Conditions of Plant Materials

All the materials including the complementation, the overexpression materials, the mutants, and other plants used in this study were grown in the experimental field of Huazhong Agricultural University in Lingshui (Hainan province, China) from December to May or in Wuhan (Hubei province, China) from May to October. The plants were subjected to normal field management.

### 4.4 Vector Construction and Generation of Various Transgenic Plants

The coding sequences of *OsGRX8*, *OsbZIP47*, and *OsbZIP08* were amplified from ZH11 cDNA by qRT‐PCR and separately cloned into the binary expression vector pCAMBIA2301U containing a maize ubiquitin promoter and a 3 × FLAG tag at the C terminal to generate the vectors *P*_Ubi_::*OsGRX8*‐FLAG, *P*_Ubi_::*OsbZIP47*‐FLAG, and *P*_Ubi_::*OsbZIP08*‐FLAG over‐expression constructs.

The fragment of a 5‐Kb sequence contains the 3‐Kb promoter region upstream of the *OsbZIP08* start codon and its 2‐Kb genomic DNA sequence was amplified from 9311 DNA and cloned into the plant binary vector pCAMBIA1300‐FLAG to generate the *P_OsbZIP08_*_‐9311_::*OsbZIP08*‐FLAG vector. A 5‐Kb fragment covers the 3‐Kb promoter region and 2‐Kb genomic sequence of *OsbZIP47* was amplified from 9311 DNA and cloned into the pCAMBIA1300‐FLAG vector to generate the *P_OsbZIP47_*_‐9311_::*OsbZIP47*‐FLAG vector. The 3‐Kb promoter region and 2‐Kb genomic sequence of *OsGRX8* was amplified from ZH11 DNA and cloned into the pCAMBIA1300‐FLAG vector to generate the *P*_OsGRX8‐ZH11_::*OsGRX8*‐FLAG vector.

The CRISPR/Cas9 gene editing technique [54] was used to obtain the mutant materials of *osbzip47*, *osbzip08*, *osbzip47*/*osbzip08*, *osgrx8*, *osgrx8/13*, and *osgrx8/13/24/26*. The designed 20 nt sgRNAs excluding the possibility of off‐target were cloned in the CRSPR/Cas9 binary expression vector to generate the CRISPR/Cas9 constructs, respectively.

All vectors were produced through homologous recombination by One‐step enzymatic assembly of DNA molecules with the In‐Fusion cloning kit (Vazyme Biotech) and confirmed by the sequencing analysis. All vectors were transformed into ZH11 or MH63 callus by *Agrobacterium tumefaciens*‐mediated transformation. The positive plants were selected by hygromycin or G418 resistance and identified by agarose or polyacrylamide gel electrophoresis of the PCR products. All the primers are shown in Table S11.

### 4.5 Phylogenetic Analysis and Protein Sequence

OsGRX8, OsbZIP47 and OsbZIP08 in rice, ROXY1, PAN in Arabidopsis and MSCA1, FEA4 in maize were used as queries to find homologs and orthologues in rice and other plant species using BLAST servers such as databases of UniProt (https://www.uniprot.org/), NCBI (http://blast.ncbi.nlm.nih.gov), and MSU (http://rice.plantbiology.msu.edu/index.shtml). All the protein sequences from organisms including Arabidopsis, maize, rice, and other organisms were collected and downloaded for protein homology analysis. Based on the obtained amino acid sequences, phylogenetic tree was constructed with the aligned protein sequences using MEGA 7.0 (http://www.megasoftware.net/index.html) and using the neighbor‐joining (NJ) method with the following parameters: Poisson correction, pairwise deletion, and bootstrap resampling 1000 times. All candidate genes were examined by domain prediction servers SMART (https://smart.embl.de/), and the alignment of protein sequences through GeneDoc 3.2 software.

### 4.6 Structure Prediction

Protein tertiary structure information is most completely embedded in the primary sequence. AlphaFold3 (https://alphafoldserver.com) and PyMOL were used to predict the structures. First, inputting the protein sequence into AlphaFold3 and opening the results to get the credibility about structure prediction, such as ipTM or pTM values. Then, using the visualization PyMOL software to observe the three‐dimension structure and find the key interaction sites among the protein complex.

### 4.7 Scanning Electron Microscopy (SEM) Examination

For electron microscopy scanning, spikelet hulls before heading were obtained and fixed in FAA (50% ethanol, 5% glacial acetic acid, and 5% formaldehyde) for 4 h. The spikelet hulls were dehydrated, dried in a critical point drier and coated gold sputter, and then imaged under a scanning electron microscope (JSM‐6390LV). Image J software was used to measure cell size and cell number.

### 4.8 Yeast Two‐hybrid Assay

Intact and various mutant versions of CDSs of *OsGRX8*, *OsbZIP47*, and *OsbZIP08* were cloned into the pGBKT7 and pGADT7 vectors, respectively, which were used as bait or prey as required. Candidate CDS fragments with restriction sites EcoRI and BamHI were fused into the pGBKT7, and with restriction sites BamHI were inserted into the pGADT7 vectors (Clontech). Specific interaction was conducted by co‐transformation of the prey and bait vectors into the yeast cells AH109 using the Gold Yeast Two‐Hybrid System (Clontech) following the manufacturer's instructions. Co‐transformants were spotted onto selective plates lacking Trp‐Leu‐His‐Ade (WLHA) and non‐selective plates lacking Trp‐Leu (WL) as controls, respectively. A bacterial colony that can grow on the WLHA solid medium well indicates protein interactions. All the primers are shown in Table S11.

### 4.9 Luciferase Complementation Assay

Full‐length CDSs of *OsGRX8*, *OsbZIP47*, and *OsbZIP08* were cloned into pCAMBIA1300‐35S‐Cluc‐RBS and pCAMBIA1300‐35S‐HA‐Nluc‐RBS vectors [55]. Site‐specific mutations were introduced into gene sequences by the PCR amplification technique to verify the function of the important amino acid sites. All vectors were transformed into *Agrobacterium* strain EHA105 as required, and then the plates were cultivated in 28‐°C incubator for 36–48 h. Bacterium cells were resuspended in infiltration buffer (10 mm MES, 10 mm MgCl2, and 100 µm acetosyringone, pH 5.6) when its concentration was enough (OD600 up to 0.6), and co‐infiltrated into 3‐week‐old tobacco (*Nicotiana benthamiana*) leaves. After genes have expressed for two days, leaves were painted with 1 mm luciferin (Promega, E1605), and luciferase signals were observed using cooled CCD‐imaging apparatus (Tanon 5200). Each assay was repeated at least three times. Primers used in this assay were listed in Table S11.

### 4.10 Pull‐Down Assay

Bacterial lysates containing GST‐*OsGRX8*, HIS‐SUMO‐*OsbZIP47*, MBP‐*OsbZIP47*, MBP‐*OsbZIP47* ^C269S^, GST‐*OsbZIP08*, MBP‐*OsbZIP08*, and MBP‐*OsbZIP08* ^C327S^ were separately obtained by recombinant protein induction and expression, and were determined by 12% SDS‐PAGE gel aiming to identify the concentration of target proteins. Bacterial lysates were mixed as required and incubated at 4‐°C for 6 h with soft rocking, respectively. Glutathione Sepharose beads (50 µL; GE Life Sciences) was added to each mixed solution, followed by incubation at 4°C for 3–4 h with gently rocking. These beads were washed six times with GST‐pull down buffer (50 mM This‐HCL, pH 7.5, 150 mm NaCl, 1 mm EDTA, pH 8.0, 1% Triton X‐100, 10% glycerol, 1 mm PMSF, and 1× Complete protease inhibitor cocktail [Roche]). Subsequently, the beads were added into the 30ul 2×SDS loading buffer and separated on 12% SDS‐PAGE gels or further detected using western blot analysis with anti‐HIS antibody (sigma; SAB5600227, 1:5000 dilution), anti‐GST antibody (Abclone; AE001, 1:5000 dilution), and anti‐MBP antibody (Abclone; AE016, 1:5000 dilution), respectively. Primers used in this assay were listed in Table S11.

### 4.11 Prokaryotic Recombinant Protein Expression and Purification

To acquire recombinant HIS‐SUMO‐*OsGRX8*, GST‐*OsGRX8*, HIS‐SUMO‐*OsbZIP47*, MBP‐*OsbZIP47*, and MBP‐*OsbZIP08*, the full‐length coding sequences of *OsGRX8* and bZIP47/08 were inserted into the prokaryotic expression vectors at the BamH I/Xho I restriction sites of PET28a‐SUMO vector, EcoR I/Xho I sites of pGEX‐4T‐1 vector, EcoR I/Sal I sites of pMAL‐cRI‐HAE vector, respectively. Every plasmid was transformed into *E. coli* BL21 (DE3) cells using chemical transformation mediated by calcium chloride. Recombinant proteins were induced for expression after culturing for 16–18 h in LB medium by the addition of 1.0 mm isopropylthio‐β‐galactoside (IPTG) with 160‐rpm shaking at 16°C and purified by Ni‐NTA resin (Thermo) for HIS‐SUMO‐tagged proteins, GST‐Sepharose beads (GE Life Sciences) for GST‐tag proteins, and amylose resin (NEW ENGLAND Biolabs, E8021S) for MBP‐tagged proteins. Protein concentration and purity were determined by 12% SDS‐PAGE gel. Primers used for these vectors were listed in Table S11.

### 4.12 BIAM‐Labelling Assay

The BIAM‐labelling assay was conducted according to described previously [29]. MBP, MBP‐OsbZIP47, MBP‐OsbZIP47^C269S^, and MBP‐OsbZIP08 proteins were expressed and purified from *E. coli* BL21 (DE3), and then were subjected to different concentrations (0 mM, 2 mm, 10 mM) of H_2_O_2_ and/or OsGRX8 as required at room temperature for 15 min under the condition of darkness. The treated proteins were precipitated by adding an equal volume of acetone, and then stewed for 20 min at −20°C. The pellets were obtained by centrifuging at 10000 × g for 5 min and washed three times using 50% acetone, and then dissolved by the addition of 500 µl labeling buffer. After incubating for 1 h, the reactions were terminated by adding 20 mM β‐mercaptoethanol, and the mixtures were precipitated by mixing one volume of acetone. The pellets were dissolved in the 30 µl 2 × SDS sample buffer and separated on SDS‐PAGE gels. Proteins labelled with BIAM were detected using biotin antibody (Cell Signaling, Cat: 7075S, 1:5000 dilution), and the total proteins of MBP, MBP‐OsbZIP47, MBP‐OsbZIP47^C269S^, and MBP‐OsbZIP08 were detected and manually kept into consistency with anti‐MBP antibody (Abclone; AE016, 1:5000 dilution).

### 4.13 ROS Content Detection

Spikelet hulls were obtained freshly using 1 × PBS buffer. And then hulls were stained using DCFH‐DA probe in 37°C for 30 min and washed 4–5 using 1 × PBS buffer. Photos were captured using a stereo fluorescence microscope. Image J software was used to measure fluorescence intensity.

### 4.14 Electrophoretic Mobility Shift Assays (EMSA)

To examine the direct binding of OsbZIP08 to the promoter of the *OsGRX8* gene, an EMSA assay was performed in this study. Single‐stranded DNA probes (∼50 bp) containing the TGACGT motif in the promoter of *OsGRX8*, were artificially synthesized with or without biotin labelled at the 5ʹ end. Double‐stranded labelled probes were incubated with purified proteins (MBP, MBP‐OsbZIP47, MBP‐OsbZIP08, and HIS‐SUMO‐OsGRX8) as required at room temperature for 20 min and at 4°C for 20 min successively, and for competition analysis, non‐labeled probes were needed to add in the reactions. DNA gel shift assays were conducted using a Chemiluminescent EMSA kit (Thermo Fisher Scientific, 20148) according to the manufacturer's instructions. The probe sequences are listed in Table S11.

### 4.15 RNA Isolation and qRT‐PCR Analysis

The expression profiles of *OsGRX8*, *OsbZIP47*, and *OsbZIP08* at different developmental stages ware downloaded from RiceXPro (https://ricexpro.dna.affrc.go.jp/Zapping/), and the drawing is showed by GraphPad.Prism.9 software.

Total RNA was extracted from the developing young panicles in different genetic backgrounds used in this paper using TRIzol RNA extraction kit (Invitrogen). Complementation DNA was synthesized from 1 ug of total RNA per sample using a reverse transcriptase kit (Vazyme) including RNAase‐free DNase I. Real‐time PCR reactions were performed with SYBR Green Mix (Vazyme) using the standard protocol of QuantStudio 6 Flex System. The *Ubiquitin* gene (*LOC_Os03g13170*) was used as the reference gene. Primers used for qRT‐PCR were listed in Table S11.

### 4.16 H_2_O_2_ and DTT Treating Assay

10‐cm panicles in the same plant of ZH11 wildtype or three *P*_*OsGRX8*‐ZH11_::*OsGRX8*‐FLAG transgenic positive plants were treated with different concentration (5 mm, 20 mm) of H_2_O_2_ or DTT, separately. The samples were acquired after treated for 0, 5, or 20 min. The RNA was extracted and qRT‐PCR analysis was conducted using the unique primer for determining the native expression level of *OsGRX8*. The *Ubiquitin* gene (*LOC_Os03g13170*) was used as the reference gene. The protein level of *OsGRX8* of these treated samples of *P*_*OsGRX8*‐ZH11_::*OsGRX8*‐FLAG transgenic plants was determined by western blotting using the Anti‐body of FLAG (Abclone; AE005, 1:5000 dilution) and anti‐Actin (Abclone; AC009, 1:5000 dilution). Primers used for qRT‐PCR were listed in Table S11.

### 4.17 Transcriptional Activity Assay

To analyze the transactivation activity of *OsbZIP47*, *OsbZIP08*, and *OsGRX8* in tobacco, the fragments of a 2‐Kb promoter *OsbZIP08* were amplified by PCR from ZH11 genomic DNA and ligated to the Sal I restriction sites of the plant binary vector pGreenII 0800‐LUC containing the *Renilla luciferase* gene as the internal control to generate the reporter vector. For the effector vectors, the coding sequence of *OsbZIP47*, *OsbZIP08*, and *OsGRX8* was inserted into the pCAMBIA 1300 vector with FLAG tag in the C terminal. The constructs were transformed separately into *Agrobacterium* strain GV3101 cells, and the plates were cultured at 28°Cfor 36–48 h until the bacterial colony was growing well. Subsequently, bacteria cells were co‐infiltrated into 3‐week‐old *N. benthamiana* leaves with the experimental group and the control group on the same leaf and expressed 2 days under the condition of an artificial illumination incubator. Tobacco samples with the same size were collected carefully and powered them in liquid nitrogen. Transactivation activity assay was performed by TECAN Infinite M200 microplate reader machine using a dual‐luciferase assay kit (Promega, E1910) according to the manufacturer's instructions. The relative LUC/REN ratios represent the ability of transactivation activity. Several independent experiments were conducted as required. Data shown as mean ± SEM (*n*>3). Statistical analyses were performed by Duncan's multiple range tests. The presence of the same lowercase letter denotes a non‐significant difference between means (*p* > 0.05). Primers were listed in Table S11.

### 4.18 ChIP‐qPCR Analysis

The ChIP‐qPCR assay using the *P_OsbZIP08_*_‐9311_::*OsbZIP08*‐FLAG transgenic plants was performed as previously described [56]. Briefly, the young panicles of *P_OsbZIP08_*_‐9311_::*OsbZIP08*‐FLAG transgenic plants after washed with PBS buffer were cross‐linked using 1% formaldehyde for 30 min, and the reaction was terminated by adding glycine, and then the samples were washed with water for two times. After the sample was powered in liquid nitrogen, and chromatin was extracted using the extraction buffer and sonicated to generate 200–500 bp DNA fragments. Immunoprecipitation was conducted using protein A/G with FLAG antibody (ABclone, AE005) and protein A/G only as the negative control at 4°C overnight. Protein DNA complexes were washed using the 0.5 x nuclei lysis buffer, high salt wash buffer, and LiCl salt wash buffer in proper order, subsequently. The pellets were eluted with the elution buffer and DNA fragments were purified and dissolved in water for further qRT‐PCR experiments. The maize *Ubi* promoter was used as another negative control. The primers used for ChIP‐qPCR are listed in Table S11.

In order to examine the effect of OsGRX8 and OsbZIP47 on OsbZIP08, we conducted the ChIP‐qPCR assays. In details, after all the panicles samples of *P_OsbZIP08_*_‐9311_::*OsbZIP08*‐FLAG, *P*_Ubi_::Flag, *P*_Ubi_::*OsGRX8*‐FLAG, and *P*_Ubi_::*OsbZIP47*‐FLAG were powered in liquid nitrogen, separately. The power of *P_OsbZIP08_*_‐9311_::*OsbZIP08*‐FLAG was divided into four parts, each part of which were mixed with equivalent samples of *P*_Ubi_::Flag, *P*_Ubi_::*OsGRX8*‐FLAG, and/or *P*_Ubi_::*OsbZIP47*‐FLAG. Chromatin was extracted using the extraction buffer for 30 min. The next steps were the same to the above description.

## Author Contributions

**X.L**. constructed part of the genetic materials, verified the relationship among *OsGRX8*, *OsbZIP47*, and *OsbZIP08* biochemically, discovered and confirmed the downstream gene *OsGRX8* of the *OsGRX8*‐(*OsbZIP47*)‐*OsbZIP08* modules, and wrote the manuscript. **M.W** finished the natural variation analysis of *OsGRX8*, *OsbZIP47*, and *OsbZIP08*, constructed most genetic materials, collected most phenotypes of the transgenic lines, and wrote the related results. **Z.Q**. assisted in determining the genotypes of all the relative materials. **J.Z**. constructed some vectors for protein interactions and finished the EMSA assays. **J.H**. and **J.X**. were involved in some experiments that verified the relationship among *OsGRX8*, *OsbZIP47*, and *OsbZIP08* biochemically. **J.Z** finished the GWAS analysis. **Y.D**. and **Y.H**. constructed some vectors for protein interactions. **X.S**. constructed the CRISPR vector for the mutant of *OsGRX8*, and assisted in finishing the genetic material. **W.L**. constructed the CRISPR vector for the double mutants of *OsbZIP47* and *OsbZIP08*, and assisted in finishing the genetic materials. Y.D. and J.Z. finished the selective sweep analyses. **Y.L**. conceptualized, supervised the research work, and wrote the manuscript.

## Conflicts of Interest

The authors declare no conflicts of interest.

## Supporting information




**Supporting File 1**: advs74109‐sup‐0001‐SuppMat.docx.

**Supporting File 2**: advs74109‐sup‐0002‐TableS1‐S11.xlsx.

## Data Availability

The data supporting the findings of this work are available within the paper and its Supplementary Information files. A reporting summary for this article is available as a Supplementary Information file. The SNP data of the mini core collection can be retrieved from http://ricevarmap.ncpgr.cn/download/. Accession codes of all genes or alleles reported in the study are available in GenBank: LOC_Os02g30850 (*OsGRX8*), [https://www.ncbi.nlm.nih.gov/nuccore/AK073461]; LOC_Os04g32300 (*OsGRX13*), [https://www.ncbi.nlm.nih.gov/nuccore/FJ463033]; LOC_Os11g43530 (*OsGRX24*), [https://www.ncbi.nlm.nih.gov/nucleotide/EE590694]; LOC_Os11g43580 (*OsGRX26*), [https://www.ncbi.nlm.nih.gov/nuccore/AK062878]; LOC_Os06g15480 (*OsbZIP47*), [https://www.ncbi.nlm.nih.gov/nuccore/AK109719.1]; LOC_Os01g59350 (*OsbZIP08*), [https://www.ncbi.nlm.nih.gov/nuccore/AK069158]. Source data are provided with this paper. The data that support the findings of this study are available from the corresponding author upon reasonable request.
