# Supplementary material for: Natural Negative Feedback Loops Confer Indica‐Japonica Differentiation for Grain Size Homeostasis in Rice
Source: Adv Sci (Weinh). 2026 Feb 4;13(19):e16180. doi: 10.1002/advs.202516180 (PMC13045434; doi:10.1002/advs.202516180)
Supplement: Supplementary file 1 — Supporting File 1: advs74109‐sup‐0001‐SuppMat.docx. [file ADVS-13-e16180-s001.docx]

**Natural negative feedback loops confer *indica*-*japonica* differentiation for grain size homeostasis in rice**

Xingxing Li^1,2,3^, Meng-en Wu^1,2,3^, Ziqi Qiao^1,2^, Junkui Huang^1,2^, Juncheng Zhang^1,2^, Yang Ding^1,2^, Junqing Zhu^1,2^, Jingyue Xu^1,2^, Yuxin Huang^1,2^, Wei Li^1,2^, Xiaomin Su^1,2^, Yue Ding^1,2^, Jianwei Zhang^1,2^ and Yibo Li^1,2^*

**Fig. S1.** Population genetic structure of the rice mini-core collection.

**Fig. S2.** GWAS of grain length, comparative expression patterns of candidate genes, important variation and haplotype network analysis of *OsGRX8*.

**Fig. S3**. Genotypes and phenotypes of transgenic lines of *OsGRX8*.

**Fig. S4.** GWAS of grain length, comparative expression profiles of candidate genes, important variation and haplotype network analysis of *OsbZIP47*.

**Fig. S5.** Phenotypes of *OsbZIP47* transgenic materials.

**Fig. S6**. GWAS of grain length, comparative expression profiles of candidate genes, important variation and haplotype network analysis of *OsbZIP08* and other phenotypes of complementary and CRISPR lines of *OsbZIP08*.

**Fig. S7**. Similar expression profiles of *OsGRX8*, *OsbZIP47* and *OsbZIP08* at different developmental stages in rice from the RiceXpro (a) and CREP (b) database.

**Fig. S8**. Phylogenetic relationship and protein domain analysis of OsGRX8 and its homologs or OsbZIP47, OsbZIP08 and their homologs in plants.

**Fig. S9**. Protein sequence alignment of OsGRX8 and its homologs, OsbZIP47, OsbZIP08 and their homologs in plants.

**Fig. S10**. Pull-down assay for the interactions of OsbZIP47 with OsbZIP47 or OsbZIP47^C269S^, and structure prediction of OsbZIP47^C17S; C269S^ homodimer, OsGRX8-OsbZIP47^C17S; C269S^ complex, OsbZIP08^C327S^ homodimer and OsGRX8-OsbZIP08 complex.

**Fig. S11**. Y2H assays for the interactions of OsGRX8, OsGRX8^C46S; 49S^ with OsbZIP47 and OsbZIP08, structure modeling for the interactions of OsGRX8 with OsbZIP47 or OsbZIP08 and flowchart of the BIAM-labelling assay.

**Fig. S12**. Structure prediction of OsbZIP47 and OsbZIP08 homodimer or heterodimer and phenotypes of the *osbzip47/osbzip08* mutants.

**Fig. S13**. Analysis of *trans*-acting factors in the *OsGRX8* promoter and EMSA assays for various binding ability.

**Fig. S14.** *OsGRX8* expression level and grain length between the differential expression of *OsbZIP47* or *OsbZIP08*, and the difference of grain length among four genotypes of *OsGRX8* and *OsbZIP08* or *OsbZIP47.*

**Fig. S15**. Genotypes and some phenotypes of *osbzip08/OsGRX8*-OE lines and primers designed for the total and native expression level of OsGRX8 by qRT-PCR.

**Fig. S16**. ROS contents in panicles of *osgrx8/13*, *osbzip08* and *P*_Actin_::OsbZIP47 lines determined by the fluorescent probe DCFH-DA.

**Fig. S17.** Correlation coefficient of the grain length and expression level of *OsGRX8*, genetic interactions between SRHs and *GS3* or *GW5* and significance of synergistic effect.

**Fig. S18.** OsGRX8 does not affect the formation of OsbZIP08 homodimer using split-LUC (a) and pull down (b) assays.


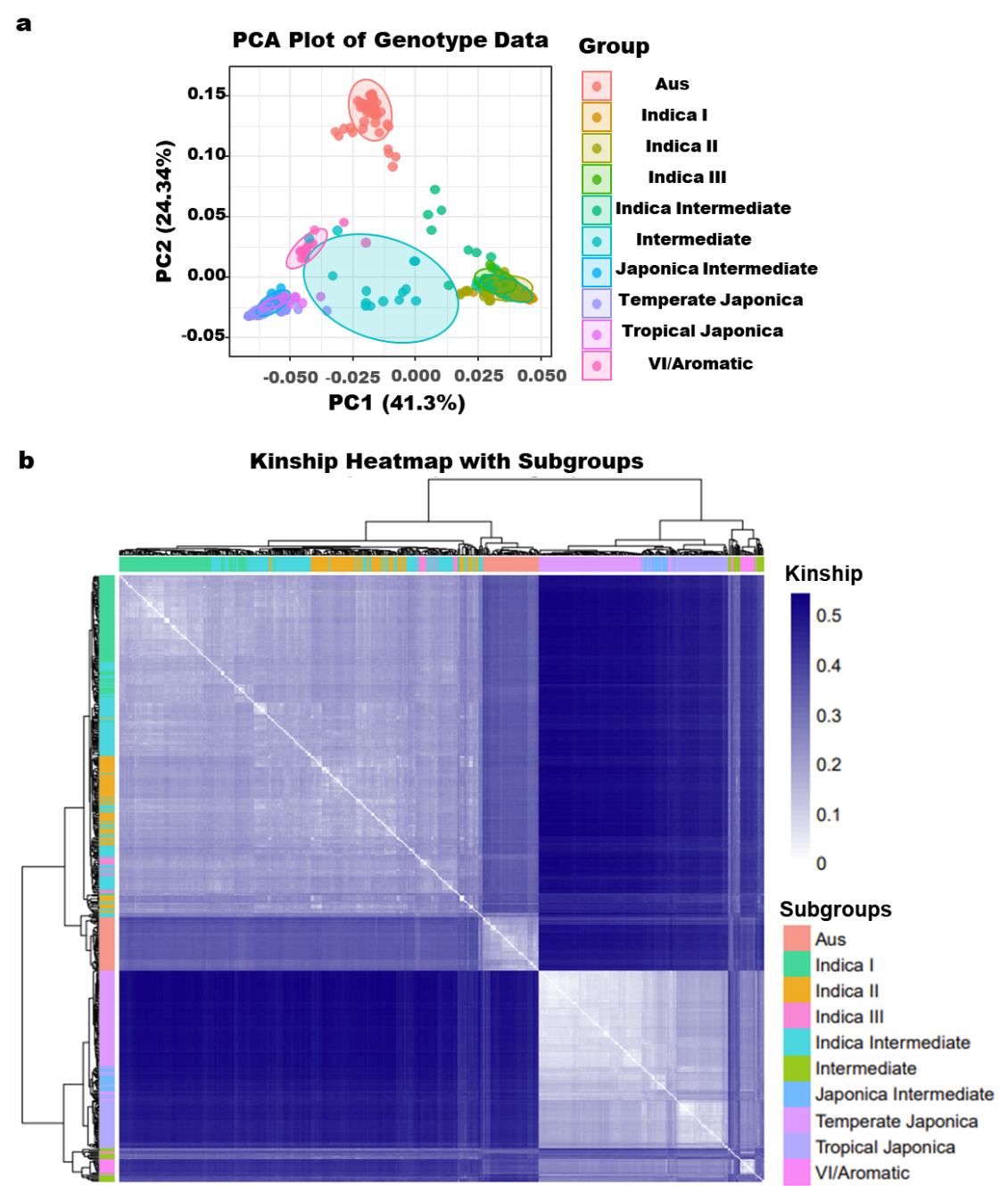


**Fig. S1. Population genetic structure of the rice mini-core collection.** (a) Principal component analysis (PCA) plot of 533 accessions, colored by subspecies. (b) Kinship heatmap depicting genetic relationships among the same set of accessions, with subgroups indicated.


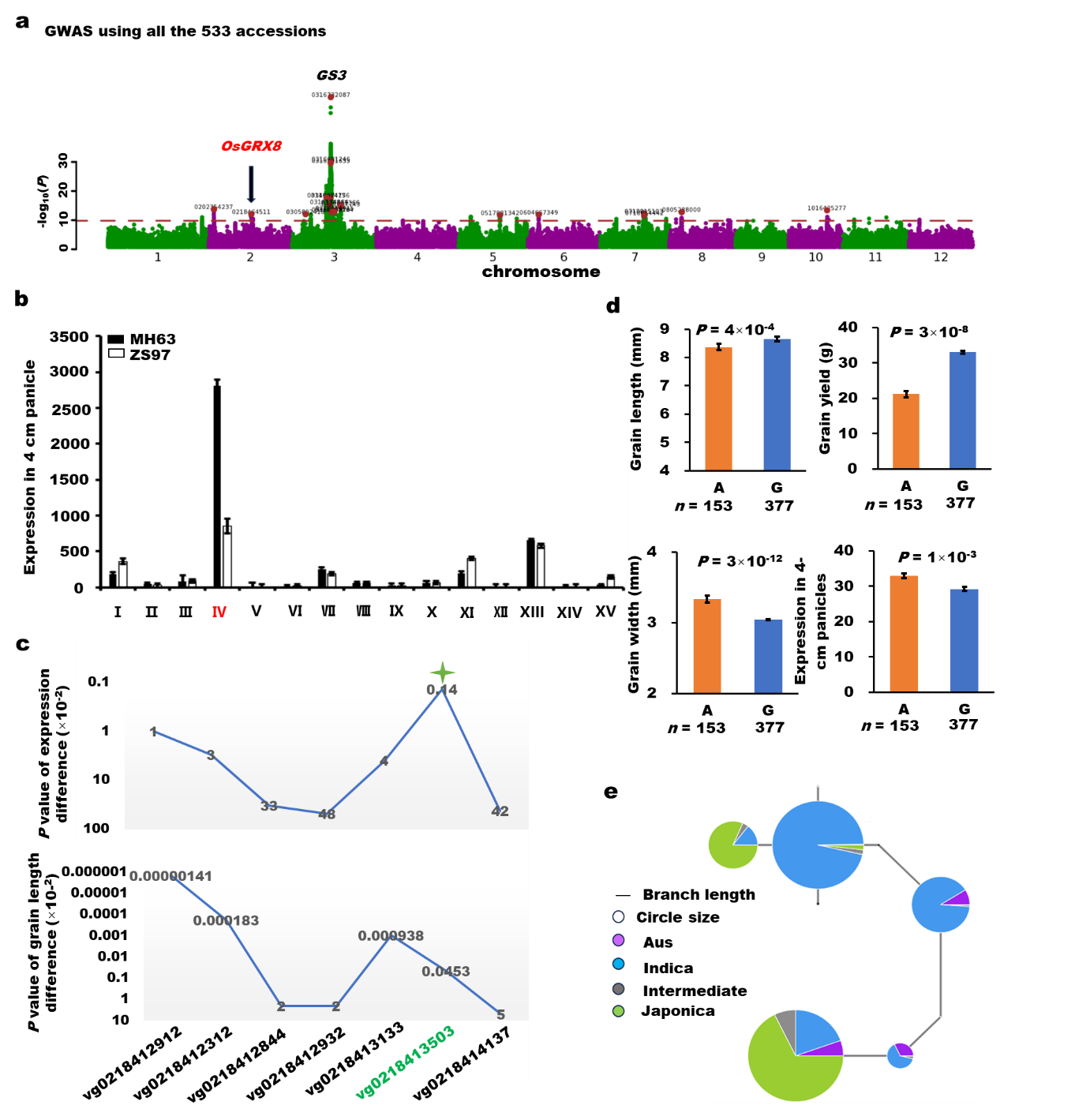


**Fig. S2. GWAS for grain length, comparative expression** **patterns of candidate genes,** **important variation and haplotype network** **analysis of** ***OsGRX8*.** **a** Manhattan of GWAS using all the 533 accessions and grain length as an indicator trait. **b** The relative expression levels of *OsGRX8* in 4-cm panicles of the two *indica* varieties Minghui 63 and Zhenshan 97 from the CREP data. **c** *P* values for grain length and expression difference of representative natural variations in the 2k promoter of *OsGRX8*. The vg0218413503 variation marked in green is the important variation. **d** Phenotypes and expression level difference of the A/G variation of *OsGRX8*. **e** Haplotype network analysis of *OsGRX8* using the representative variations of its promoter and coding region in 4726 rice accessions.


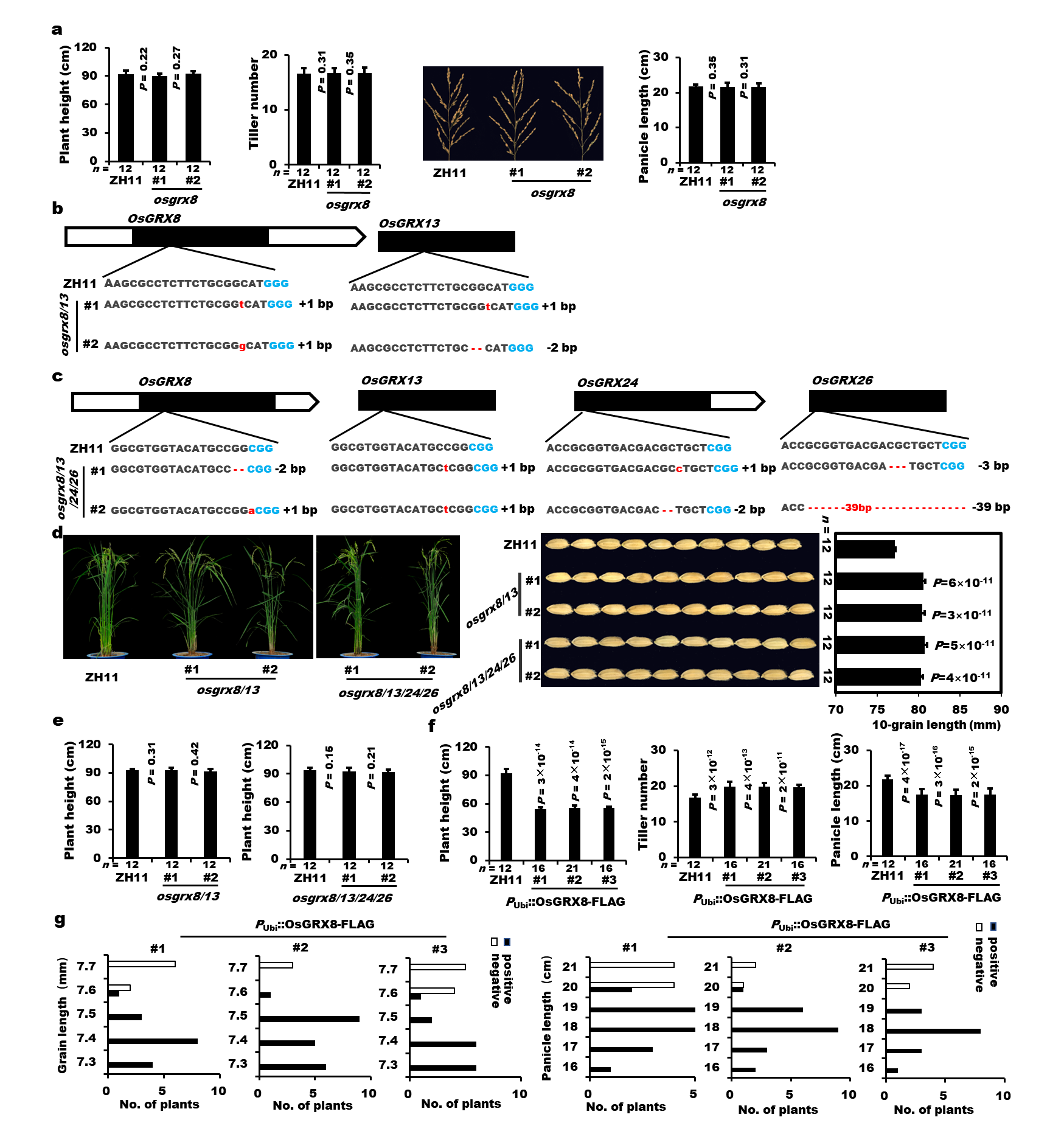


**Fig. S3. Genotypes and phenotypes of transgenic lines of *OsGRX8*. a** Plant height, tiller number and panicle morphologies in T_1_ *osgrx8* mutant lines. **b, c** The sgRNA target site and genotypes of the two independent *osgrx8/13* **(b)** or *osgrx8/13/24/26* **(c)** lines obtained by the CRISPR-Cas9 technology in ZH11. **d, e** Plant architecture and grain length of *osgrx8/13* **(d)** and *osgrx8/13/24/26* **(e)** mutant lines in T_2_. **f** Plant height, tiller number and panicle length of overexpression lines of *OsGRX8* in T_1_ progenies. **g** Co-segregation tests of grain-length and panicle-length phenotype of overexpression lines of *OsGRX8* in T_1_ progenies. PAM sequences are marked in blue. *n* is the number of accessions of each haplotype or individuals of each transgenic line. All data are shown as mean value +/- SEM. All the *P* values were produced by the two-tailed *t*-tests.


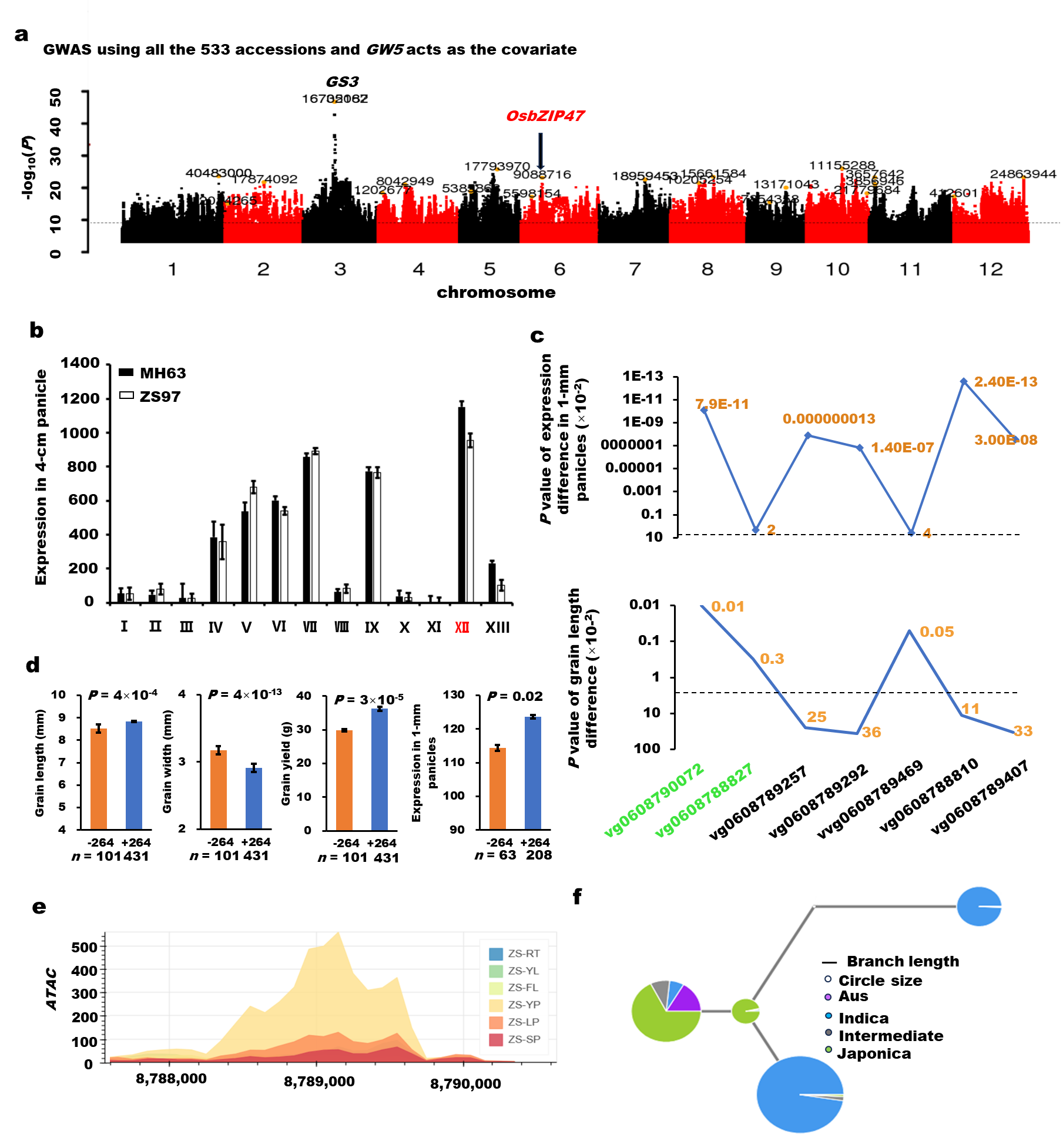


**Fig. S4. GWAS of grain length, comparative expression profiles of candidate genes,** **important variation and haplotype network analysis of *OsbZIP47*.** **a** Manhattan of GWAS using all the 533 accessions with *GW5* as the covariate and grain length as an indicator trait. **b** The relative expression levels of *OsbZIP47* in 4-cm panicles of the two *indica* varieties Minghui 63 and Zhenshan 97 from the CREP data. **c** *P* values for grain length and expression difference of representative natural variations in the *OsbZIP47* promoter. **d** Grain length, grain width, grain yield and expression levels of the vg0608788827 variation. **e** Chromatin accessible regions in the promoter of *OsbZIP47* from the RiceVarMap v2.0 database. The data for chromatin accessibility profiles contains six tissues (root/ZS-RT, young leaf/ZS-YL, flag leaf/ZS-FL, young panicle/ZS-YP, lemma & palea/ZS-LP, and stamen & pistil/ZS-SP) of Zhenshan97 measured by the ATAC-seq assay. **f** Haplotype network analysis of *OsbZIP47* using the representative variations of its promoter and coding region in 4726 rice accessions.


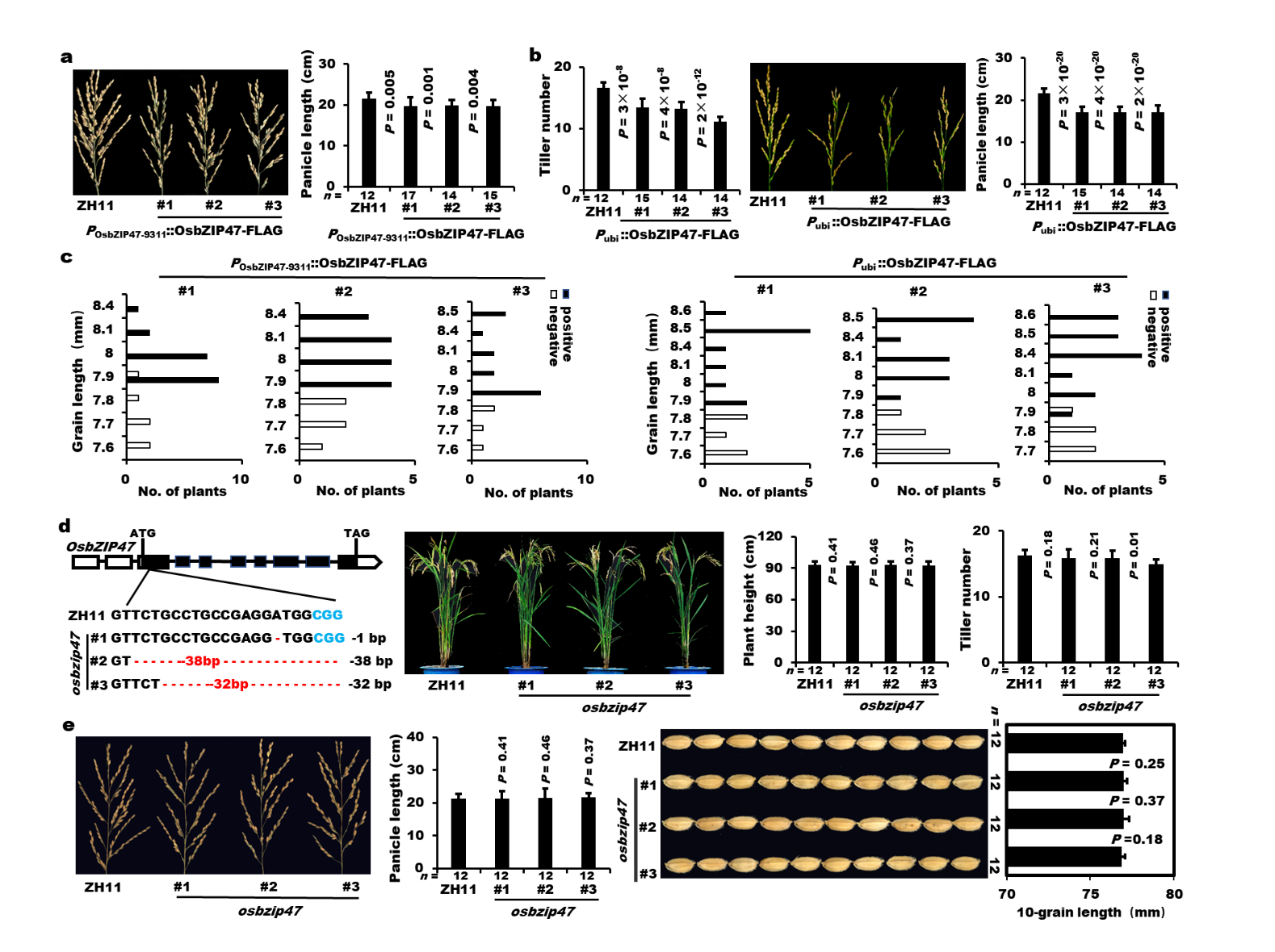


**Fig. S5. Phenotypes of *OsbZIP47* transgenic materials. a** Panicle morphologies of *P*_OsbZIP47-9311_::OsbZIP47-FLAG complementation lines in T_1_. **b** Tiller number and panicle morphologies of three *P*_Ubi_::OsbZIP47-FLAG transgenic lines in T_1_. **c** Co-segregation tests of genotype and grain-length phenotype in the three independent complementary T_1_ lines of *P*_OsbZIP47-9311_::OsbZIP47-FLAG and *P*_Ubi_::OsbZIP47-FLAG materials. **d** Plant architecture of three independent CRISPR/Cas9 lines of *OsbZIP47* in ZH11. Blue fonts represent the PAM sequence. **e** Panicle morphology and grain size in T_1_ progenies of mutant lines of *OsbZIP47*. *n* is the number of accessions of each haplotype or individuals of each transgenic line. All data are shown as mean value +/- SEM. All the *P* values were produced by the two-tailed *t*-tests.


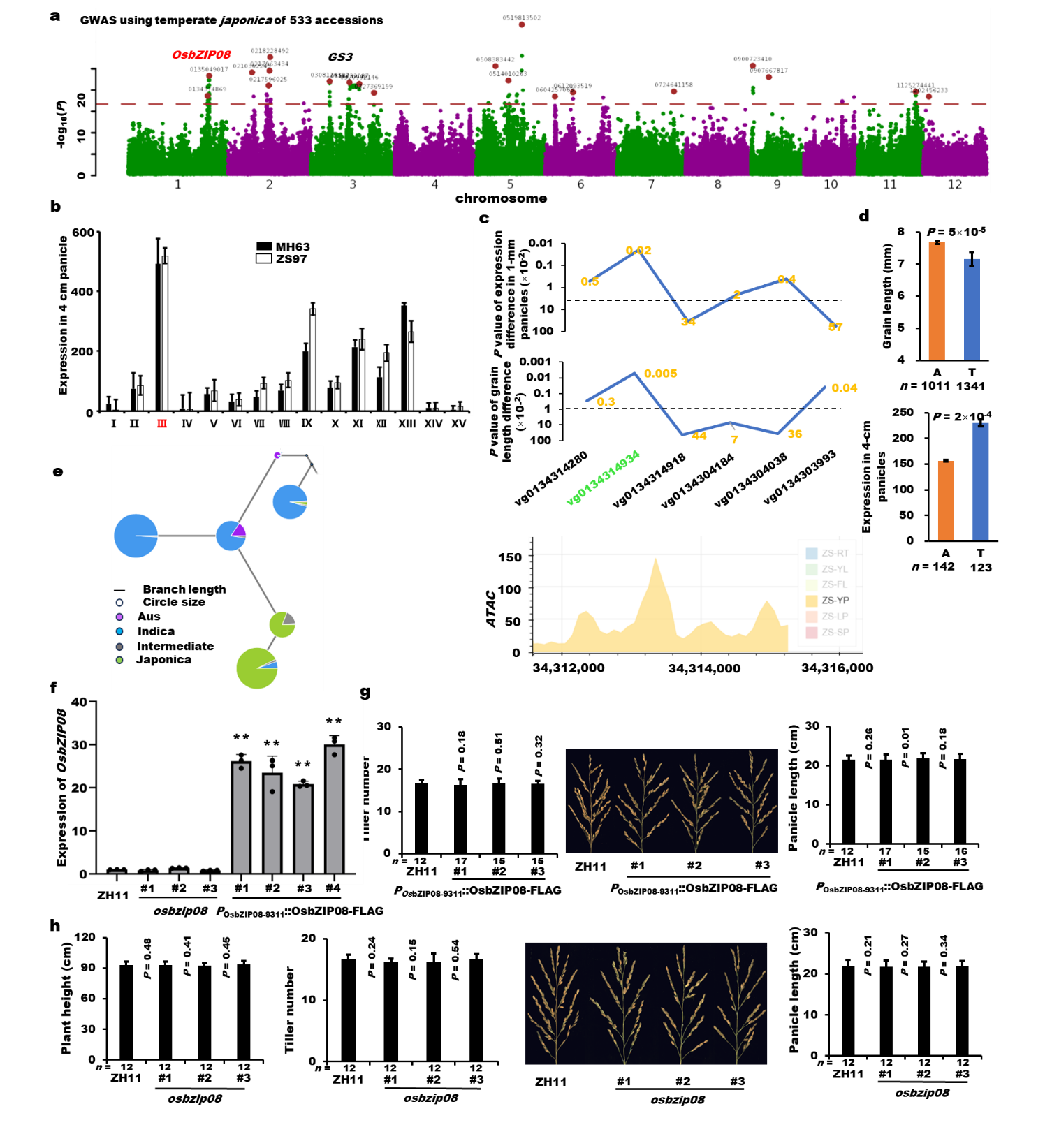


**Fig. S6. GWAS of grain length, comparative expression profiles of candidate genes, important variation and haplotype network analysis of *OsbZIP08* and other phenotypes of complementary and CRISPR lines of *OsbZIP08***. **a** Manhattan of GWAS using temperate *japonica* in 533 accessions and grain length as an indicator trait. **b** The relative expression levels of *OsbZIP08* in 4-cm panicles of the two *indica* varieties Minghui 63 and Zhenshan 97 from the CREP data. **c** *P* values for grain length and expression difference of the representative natural variations and chromatin accessible regions in the *OsbZIP08* promoter. **d** Grain length, plant height, 1000-grain weight and expression levels of the vg0134314934 variation. **e** Haplotype network analysis of *OsbZIP08* using the representative variations of its promoter and coding region in 4726 rice accessions. **f** Expression of *OsbZIP08* in ZH11, *osbzip08* and *P*_OsbZIP08-9311_::OsbZIP08-FLAG lines determined by RT-qPCR analysis. **g** Tiller number and panicle morphology in T_1_ progenies of complementary lines of *OsbZIP08*. **h** Plant architecture, tiller number and panicle morphology of three independent CRISPR/Cas9 lines of *OsbZIP08* in ZH11. *n* is the number of accessions of each haplotype or individuals of each transgenic line. All data are shown as mean value +/- SEM. All the *P* values were produced by the two-tailed *t*-tests.


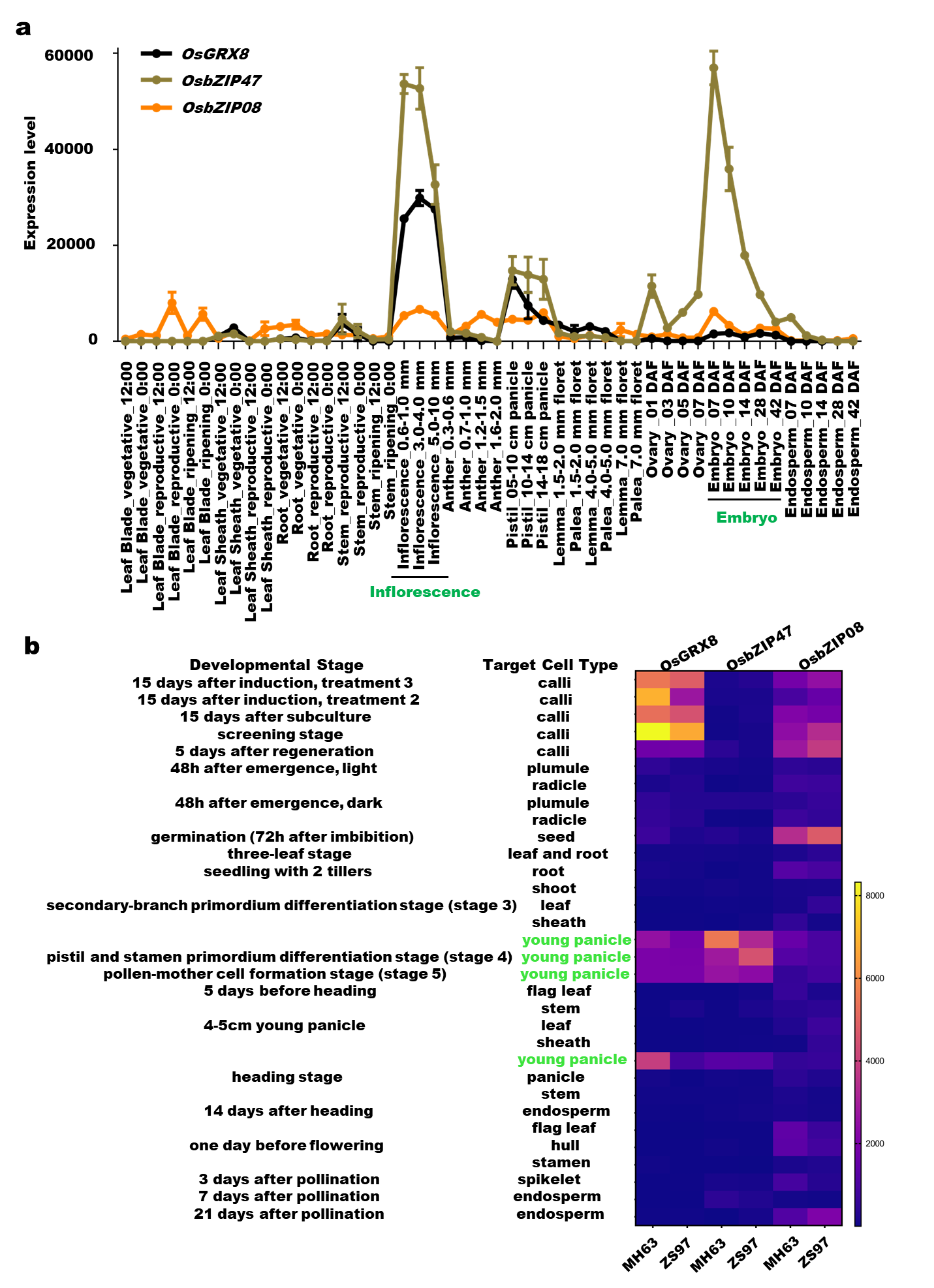


**Fig. S7. Similar expression profiles of *OsGRX8*, *OsbZIP47* and *OsbZIP08* at different developmental stages in rice from the RiceXpro (a) and CREP (b) database.**


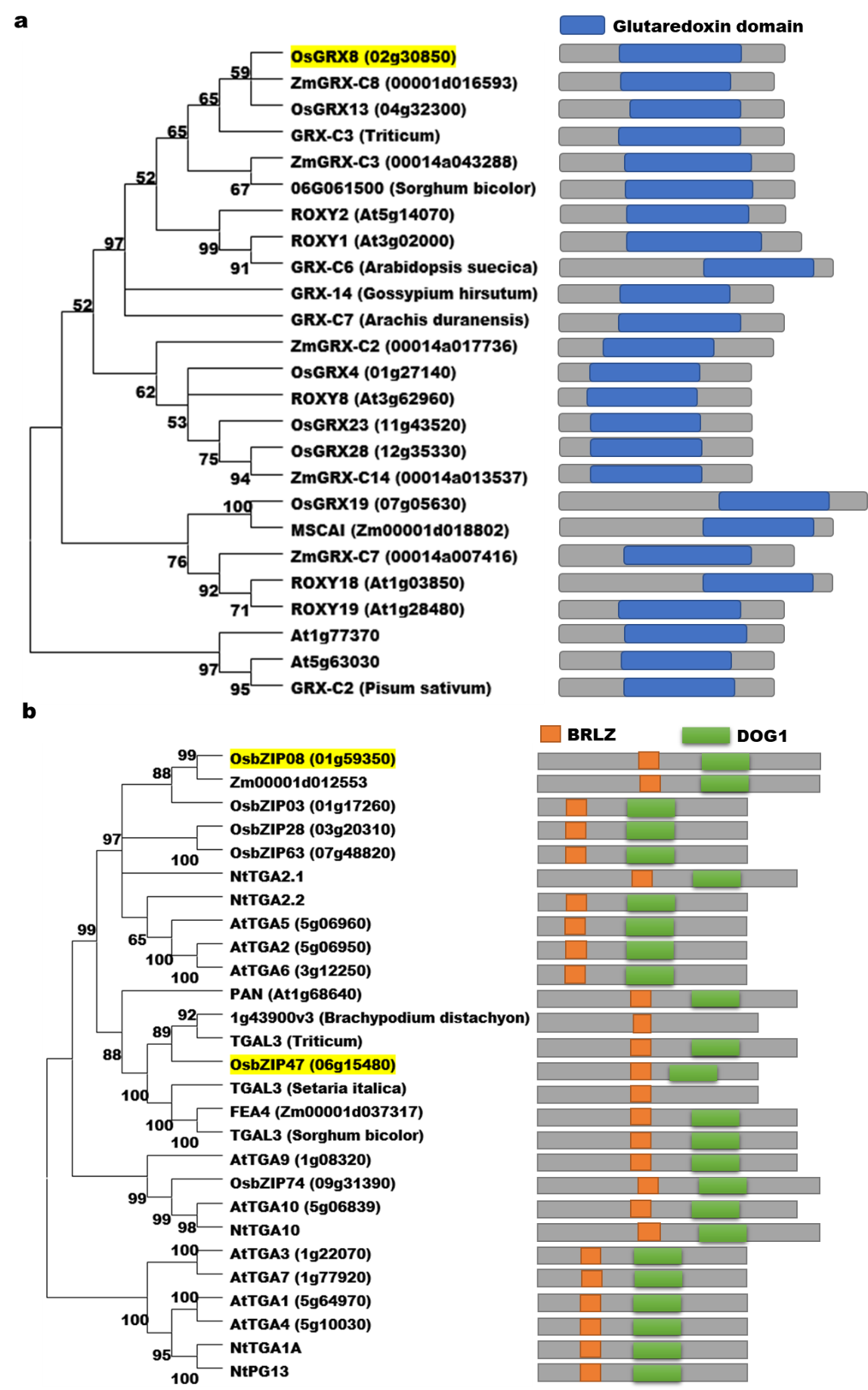


**Fig. S8. Phylogenetic relationship and protein domain analysis of OsGRX8 and its homologs or OsbZIP47, OsbZIP08 and their** **homologs in plants. a** Phylogenetic tree analysis and protein domain analysis of OsGRX8 and their homologs. **b** Phylogenetic tree analysis and protein domain analysis of OsbZIP47, OsbZIP08 and their homologs.


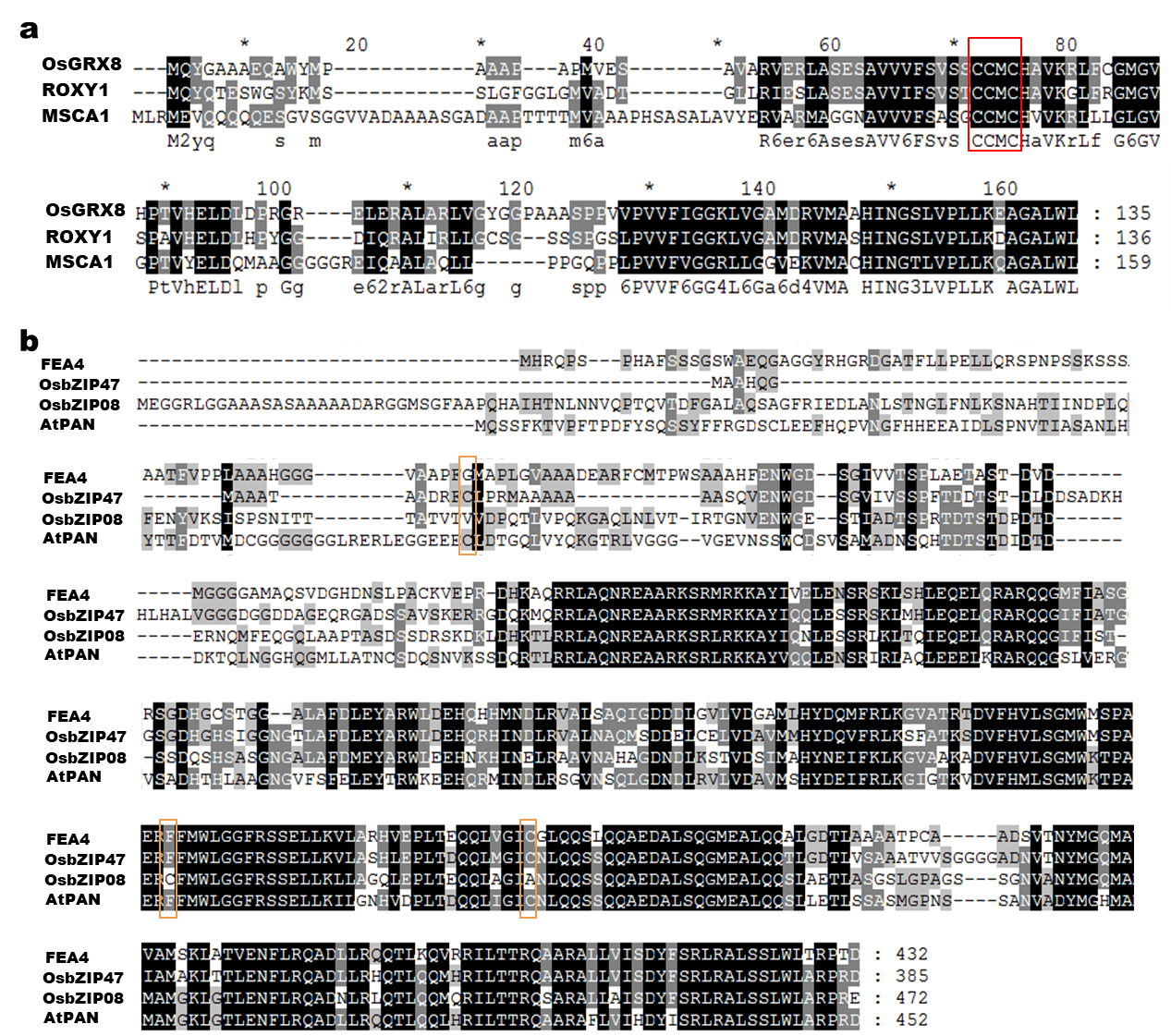


**Fig. S9. Protein sequence alignment of** **OsGRX8 and its homologs, OsbZIP47, OsbZIP08 and others in plants. a** Protein sequence alignment of OsGRX8 in rice with ROXY1 in *Arabidopsis* and MSCA1 in maize. The conserved CCMC domain was marked in red box. **b** Protein sequence alignment of OsbZIP47 and OsbZIP08 in rice with AtPAN in *Arabidopsis* and FEA4 in maize. The yellow boxes indicated the key cysteine residues.


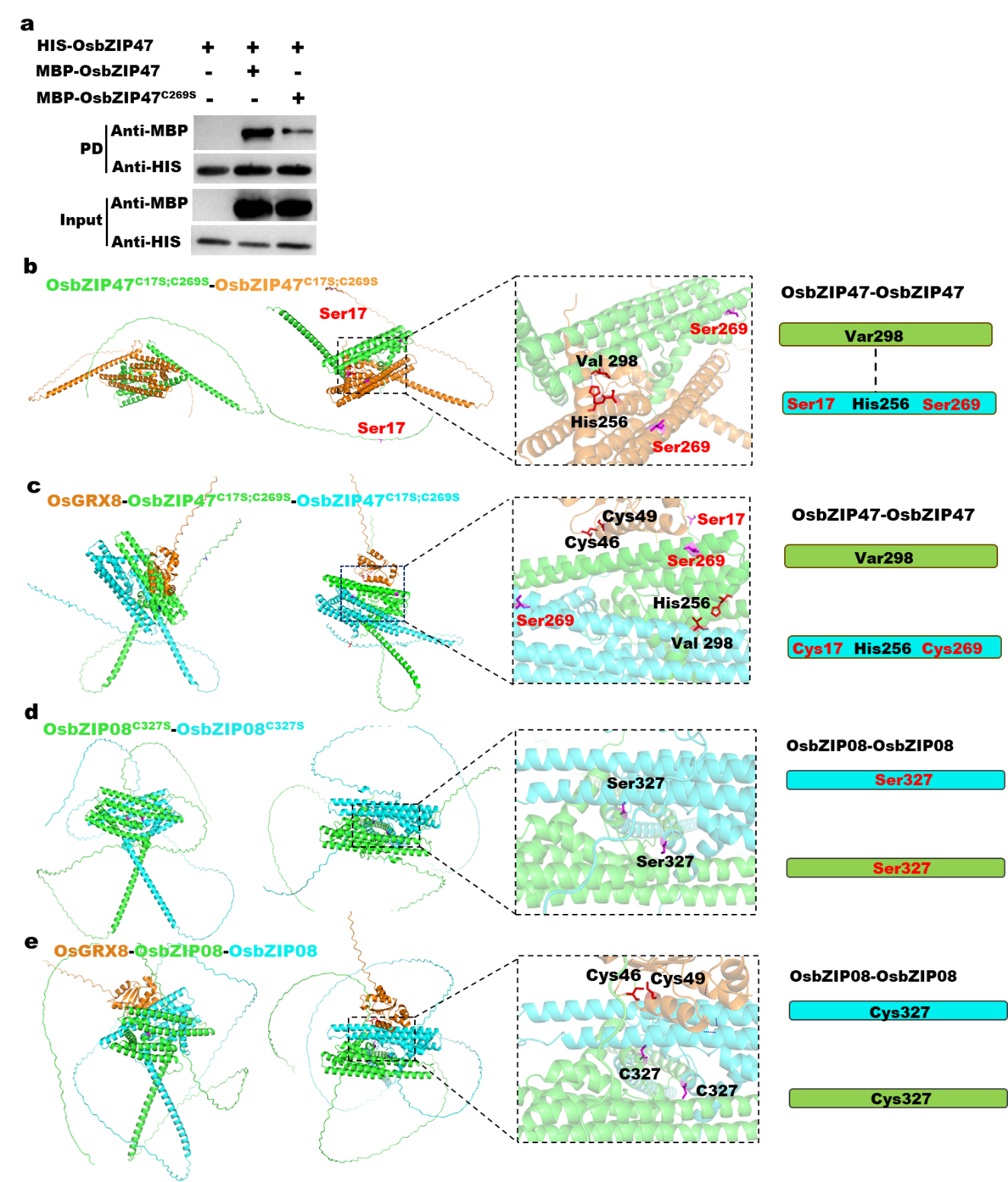


**Fig. S10. Pull-down assay for the interactions of OsbZIP47 with OsbZIP47 or OsbZIP47^C269S^, and structure prediction of OsbZIP47^C17S; C269S^ homodimer,** **OsGRX8- OsbZIP47^C17S; C269S^ complex, OsbZIP08^C327S^ homodimer and OsGRX8-OsbZIP08 complex. a** Pull-down assays for the interactions of OsbZIP47 with OsbZIP47 or OsbZIP47^C269S^. **b–e** Protein structure prediction of OsbZIP47^C17S; C269S^ homodimer (**b**), OsGRX8-OsbZIP47^C17S; C269S^ complex (**c**), OsbZIP08^C327S^ homodimer (**d**) and OsGRX8-OsbZIP08 complex (e) using AalphaFold3 and visualization software PyMOL. Intermolecular force was showed using dotted line.


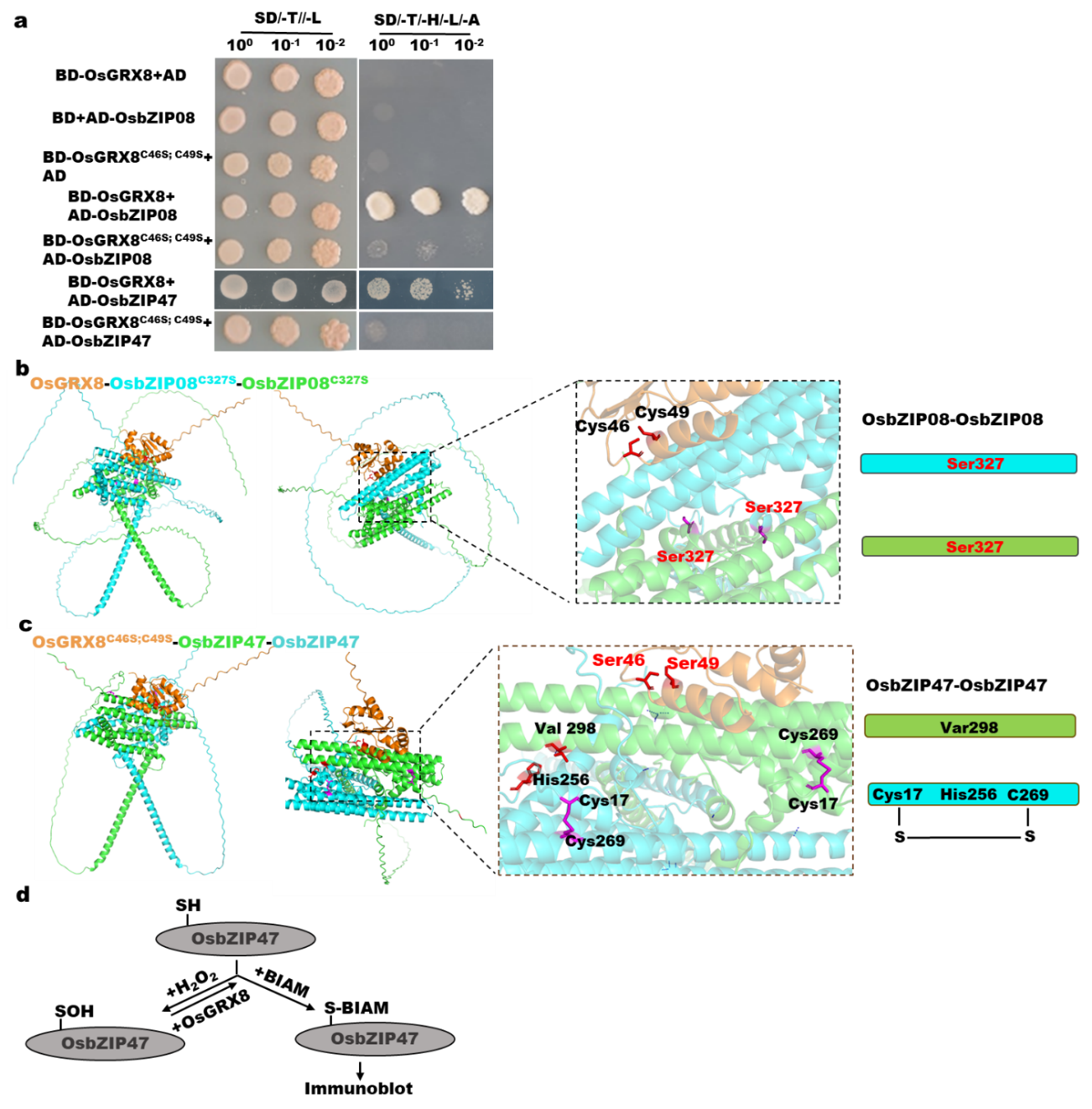


**Fig. S11. Y2H assays for the interactions of OsGRX8, OsGRX8^C46S; 49S^ with OsbZIP47 and OsbZIP08, structure** **modeling for the interactions of OsGRX8 with OsbZIP47 or OsbZIP08 and flowchart of the BIAM-labelling assay. a** Y2H assays for the interactions of OsGRX8, OsGRX8^C46S; 49S^ with OsbZIP47 and OsbZIP08. **b, c** Structure modeling for the interactions of OsGRX8 and OsbZIP08^C327S^ complex (**b**) or OsGRX8^C46S; C49S^ and OsbZIP47 complex (**c**) using AalphaFold3 and visualization software PyMOL. Intramolecular disulfide bond was marked in solid line. **d** Flowchart of the BIAM-labelling assay. Using BIAM-labelling assay to investigate whether OsbZIP47 and OsbZIP08 contains redox-sensitive cysteine residues and can be modified by OsGRX8. S-BIAM represents the thiol labelled with biotin.


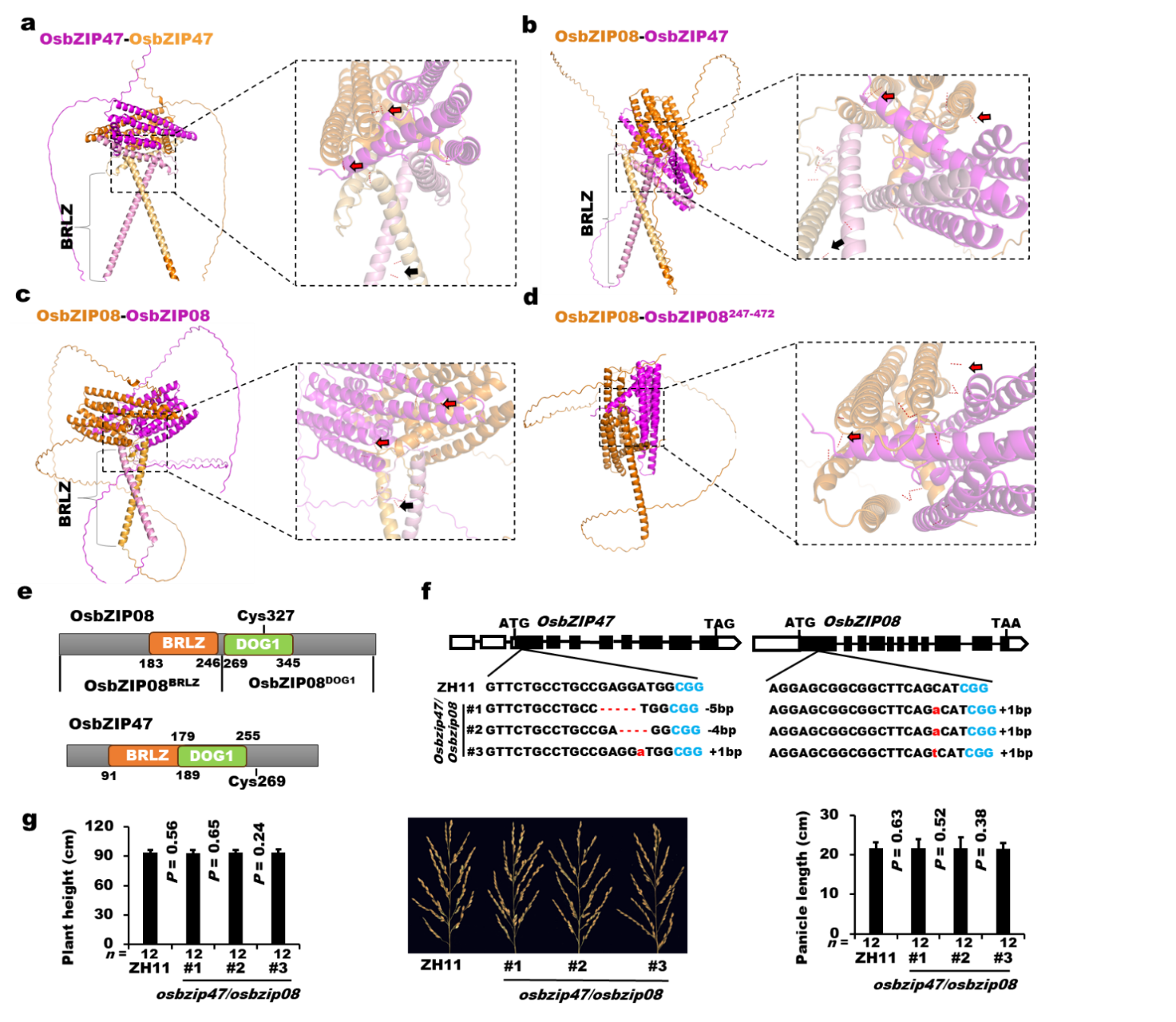


**Fig. S12. Structure prediction of OsbZIP47 and OsbZIP08 homodimer or heterodimer and phenotypes of the *osbzip47/osbzip08* mutants. a–d** Structure prediction of OsbZIP47 homodimer (**a**) and OsbZIP47/OsbZIP08 heterodimer (**b**), OsbZIP08 homodimer (**c**) and OsbZIP08-OsbZIP08^247–472^ using AalphaFold3 and visualization software PyMOL. Blank arrows represented the interactive force in BRLZ domain, and the red arrows represented the interactive force in DOG1 domain. **e** BRLZ and DOG1 domains of OsbZIP08 and OsbZIP47 were marked. **f** Genotypes of the three independent double mutants of *OsbZIP47* and *OsbZIP08* obtained by the CRISPR-Cas9 technology. **g** Plant height and panicle morphology of the three independent *osbzip47/osbzip08* double mutant lines compared to ZH11 in T_2_ progenies. All the *P* values were produced by the two-tailed *t*-tests.


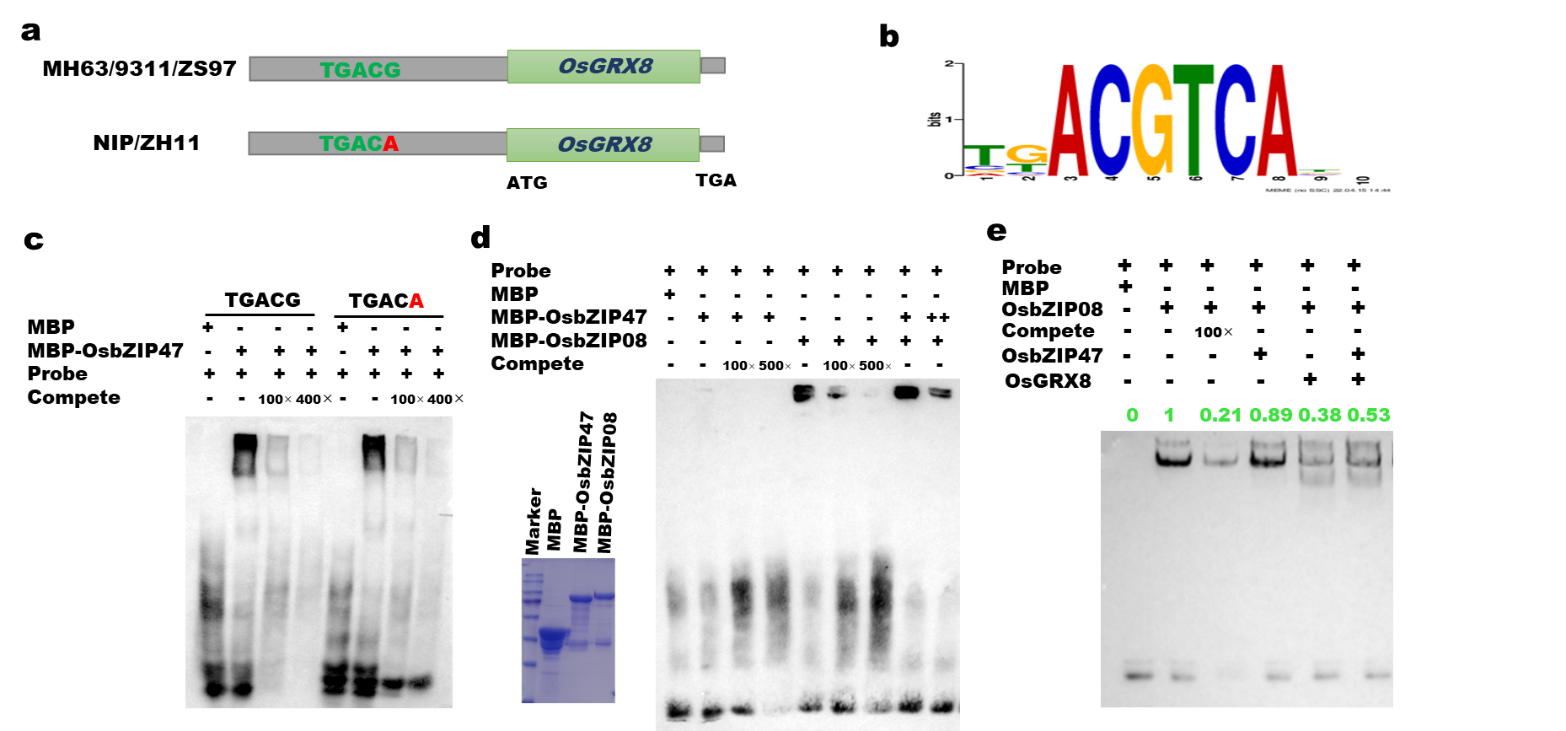


**Fig. S13. Analysis of *trans*-acting factors in the *OsGRX8* promoter and EMSA assays for various binding ability. a** Sequence comparison of the five kinds varieties, including MH63, 9311, ZS97 *indica* varieties and NIP, ZH11 *japonica* varieties. The vg0218413503 is marked in green and red. **b** The PlantPAN 2.0 was used to identify *cis*-acting elements and trans-acting factor. **c** EMSA assays checked the similar binding ability of OsbZIP47 on TGACG and TGACA motifs from *indica* and *japonica* promoters of *OsGRX8*, respectively. **d** EMSA assays for the binding ability of OsbZIP47 and OsbZIP08 on TGACA motif and the binding difference of OsbZIP08 affected by OsbZIP47. MBP-OsbZIP08 and MBP-OsbZIP47 confused proteins were purified and their concentration and purity were detected by SDS-PAGE (left). **e** EMSA assays for the binding ability of OsbZIP08 affected by OsbZIP47, OsGRX8 or OsGRX8 and OsbZIP47. The binding extent was quantified using image J.


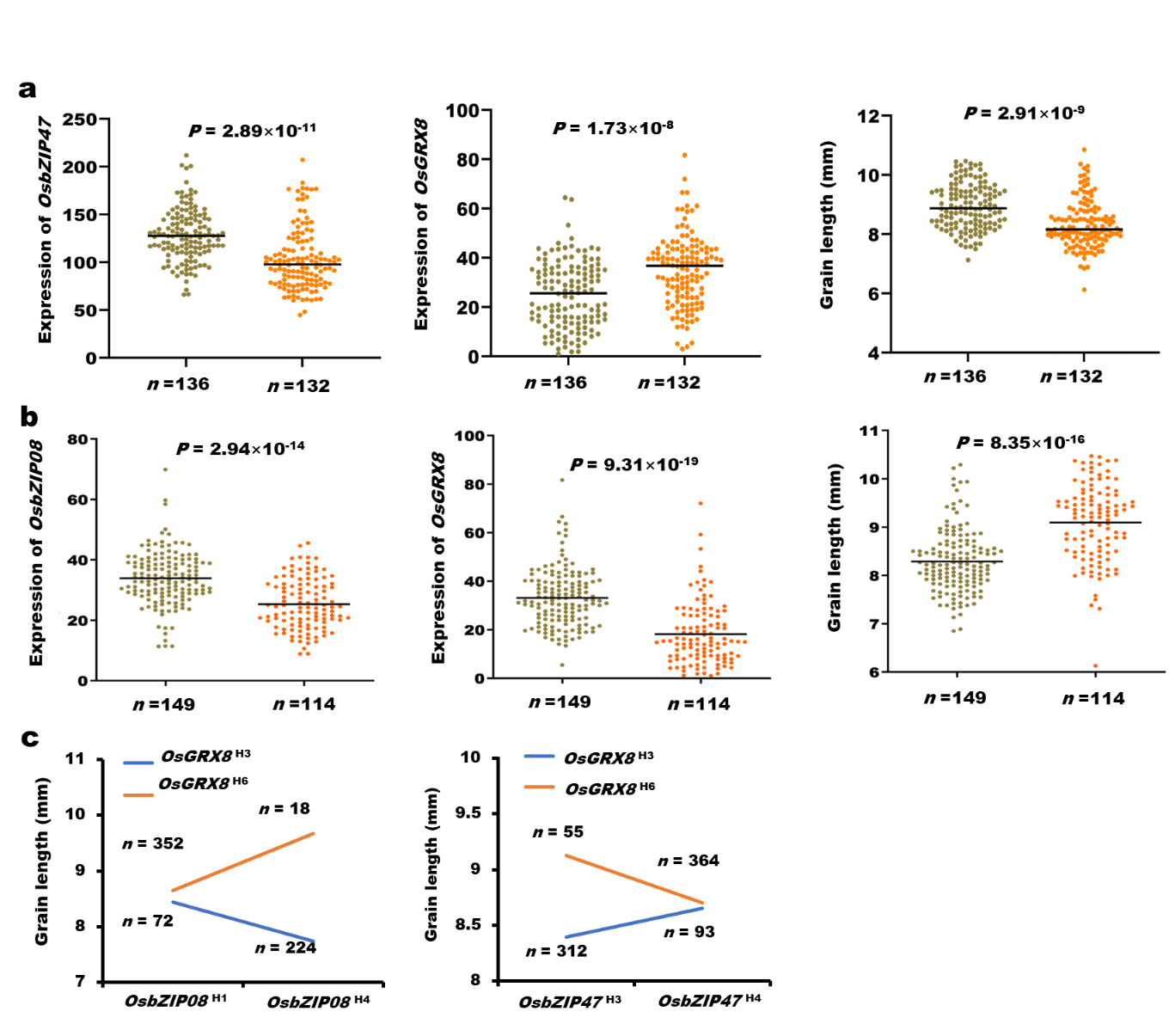


**Fig. S14.** ***OsGRX8* expression level and grain length between the differential expression of *OsbZIP47* or *OsbZIP08*, and the difference of grain length among four genotypes of *OsGRX8* and *OsbZIP08* or *OsbZIP47.* a** Differential expression of *OsGRX8* and grain-length phenotype between the 136 accessions with high expression of *OsbZIP47* and 132 accessions with low expression of *OsbZIP47* in the rice mini-core collection of 271 accessions. **b** Differential expression of *OsGRX8* and grain-length phenotype between the 149 accessions with high expression of *OsbZIP08* and 114 accessions with low expression of *OsbZIP08* in the rice mini-core collection of 271 accessions. **c** The difference of grain length phenotypes among four genotypes of *OsGRX8* and *OsbZIP08* or *OsbZIP47* in 2013 accessions, respectively. All the *P* values were produced by the two-tailed *t*-tests.


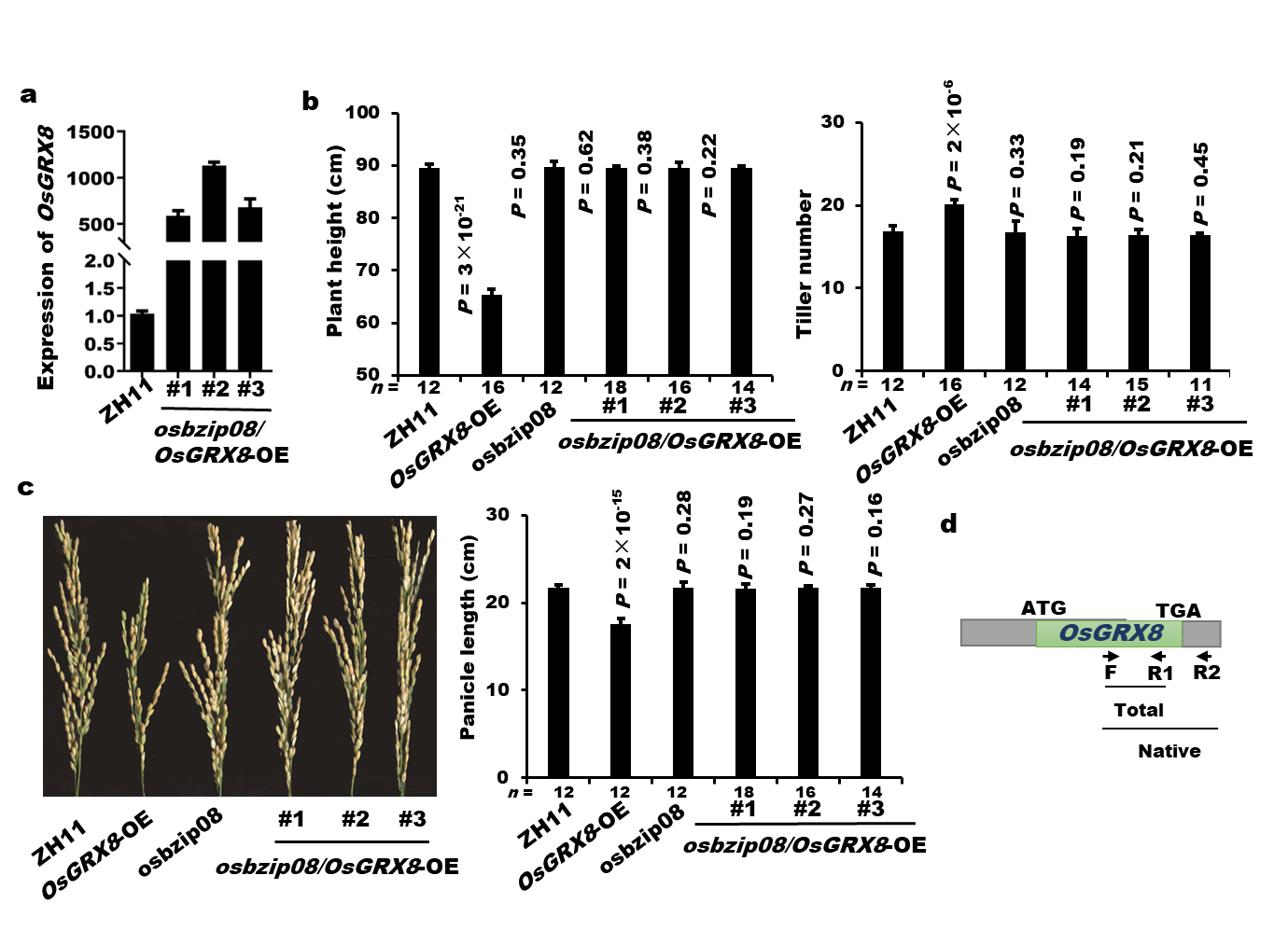


**Fig. S15. Genotypes and some phenotypes of *osbzip08/OsGRX8*-OE lines** **and** **primers designed for the total and native expression level of *OsGRX8* by qRT-PCR. a** Expression level of the *osbzip08/OsGRX8-OE* lines. **b, c** Plant architecture (**b**) and panicle morphologies (**c**) of WT(ZH11), *OsGRX8*-OE, *osbzip08*, and *osbzip08/OsGRX8*-OE lines. *n* is the number of accessions or individuals of each transgenic line. All data are shown as mean value +/- SEM. All the *P* values were produced by two-tailed *t*-tests. **d** Total and Native are the specific primers for qRT-PCR to determine the total and native expression level of *OsGRX8* in *OsGRX8* transgenic plants, respectively.


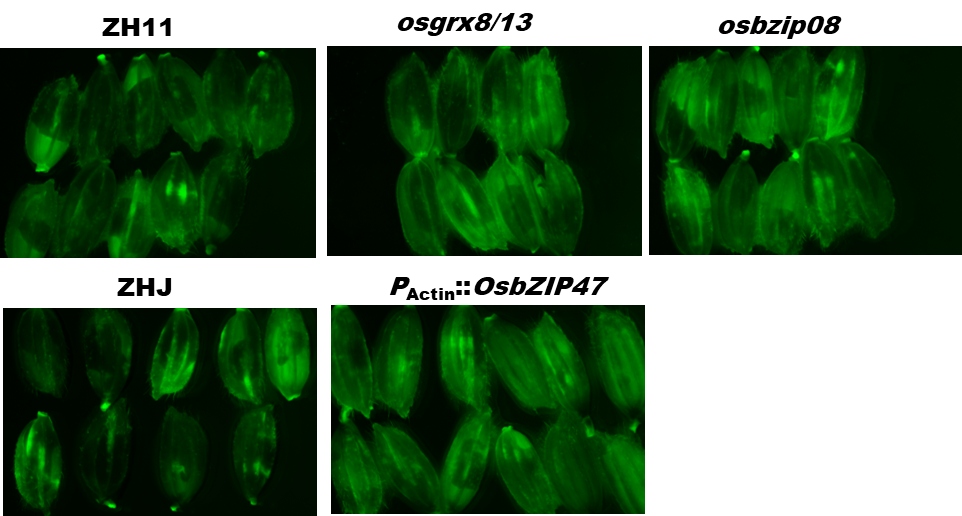


**Fig. S16. ROS contents in panicles of *osgrx8/13*, *osbzip08* and *P*_Actin_::OsbZIP47 lines determined by the fluorescent probe DCFH-DA.**


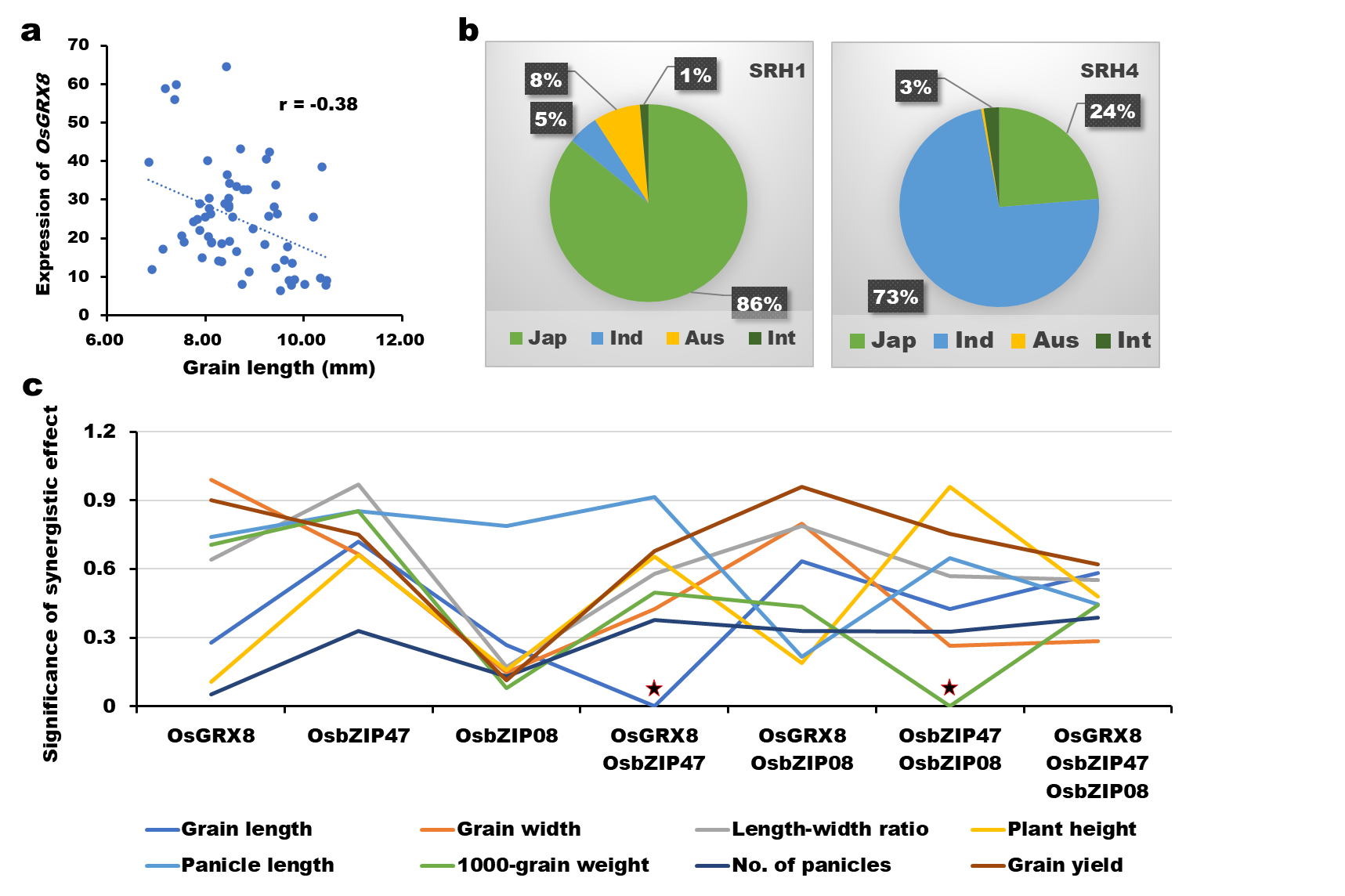


**Fig. S17 Correlation coefficient of the grain length and expression level of *OsGRX8*, genetic interactions between SRHs and *GS3* or *GW5* and significance of synergistic effect. a** Correlation coefficient of the grain length and expression level of *OsGRX8* in 31 *japonica* accessions. **b** The two major SRHs, SRH1 and SRH4, are predominant in *japonica* (85.9%) and *indica* (73.4%) subspecies in 4726 accessions. **c** The significances of synergistic effect among *OsbZIP47*, *OsbZIP08*, *OsGRX8* on the important yield traits.

**
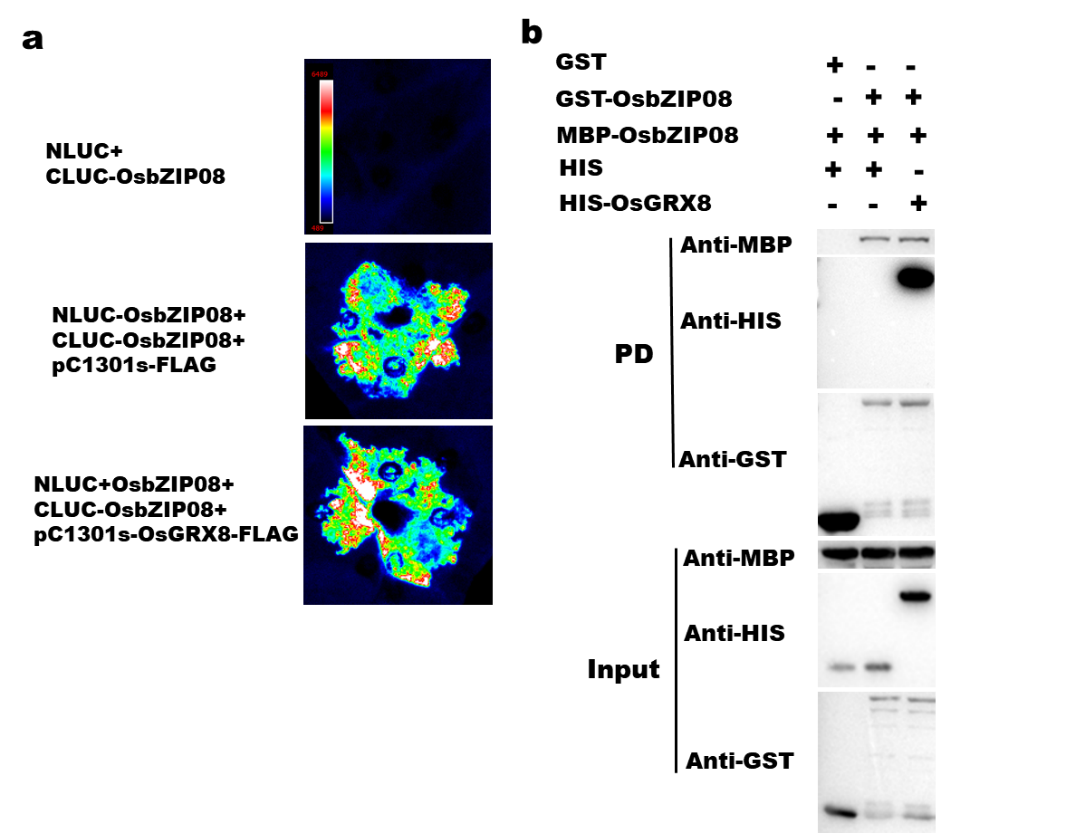
**

**Fig. S18. OsGRX8 does not affect the formation of OsbZIP08 homodimer** **using split-LUC (a) and pull down (b) assays.**
